# Resident cell lineages are preserved in pulmonary vascular remodeling

**DOI:** 10.1002/path.5044

**Published:** 2018-03-09

**Authors:** Slaven Crnkovic, Leigh M Marsh, Elie El Agha, Robert Voswinckel, Bahil Ghanim, Walter Klepetko, Elvira Stacher‐Priehse, Horst Olschewski, Wilhelm Bloch, Saverio Bellusci, Andrea Olschewski, Grazyna Kwapiszewska

**Affiliations:** ^1^ Ludwig Boltzmann Institute for Lung Vascular Research Graz Austria; ^2^ Department of Physiology Medical University of Graz Graz Austria; ^3^ Excellence Cluster Cardio‐Pulmonary System (ECCPS), Member of the German Center for Lung Research (DZL), Universities of Giessen and Marburg Lung Center (UGMLC) Justus Liebig University Giessen Giessen Germany; ^4^ Clinic for Internal Medicine Friedberg Hospital Friedberg Germany; ^5^ Department of Thoracic Surgery Medical University of Vienna Vienna Austria; ^6^ Institute of Pathology Medical University of Graz Graz Austria; ^7^ Department of Internal Medicine, Division of Pulmonology Medical University of Graz Graz Austria; ^8^ German Sports University Cologne Cologne Germany

**Keywords:** fate mapping, pulmonary vascular remodeling

## Abstract

Pulmonary vascular remodeling is the main pathological hallmark of pulmonary hypertension disease. We undertook a comprehensive and multilevel approach to investigate the origin of smooth muscle actin‐expressing cells in remodeled vessels. Transgenic mice that allow for specific, inducible, and permanent labeling of endothelial (Cdh5‐tdTomato), smooth muscle (Acta2‐, Myh11‐tdTomato), pericyte (Cspg4‐tdTomato), and fibroblast (Pdgfra‐tdTomato) lineages were used to delineate the cellular origins of pulmonary vascular remodeling. Mapping the fate of major lung resident cell types revealed smooth muscle cells (SMCs) as the predominant source of cells that populate remodeled pulmonary vessels in chronic hypoxia and allergen‐induced murine models. Combining in vivo cell type‐specific, time‐controlled labeling of proliferating cells with a pulmonary artery phenotypic explant assay, we identified proliferation of SMCs as an underlying remodeling pathomechanism. Multicolor immunofluorescence analysis showed a preserved pattern of cell type marker localization in murine and human pulmonary arteries, in both donors and idiopathic pulmonary arterial hypertension (IPAH) patients. Whilst neural glial antigen 2 (chondroitin sulfate proteoglycan 4) labeled mostly vascular supportive cells with partial overlap with SMC markers, PDGFRα‐expressing cells were observed in the perivascular compartment. The luminal vessel side was lined by a single cell layer expressing endothelial markers followed by an adjacent and distinct layer defined by SMC marker expression and pronounced thickening in remodeled vessels. Quantitative flow cytometric analysis of single cell digests of diverse pulmonary artery layers showed the preserved separation into two discrete cell populations expressing either endothelial cell (EC) or SMC markers in human remodeled vessels. Additionally, we found no evidence of overlap between EC and SMC ultrastructural characteristics using electron microscopy in either donor or IPAH arteries. Lineage‐specific marker expression profiles are retained during pulmonary vascular remodeling without any indication of cell type conversion. The expansion of resident SMCs is the major underlying and evolutionarily conserved paradigm of pulmonary vascular disease pathogenesis. © 2018 The Authors. *The Journal of Pathology* published by John Wiley & Sons Ltd on behalf of Pathological Society of Great Britain and Ireland.

## Introduction

Pulmonary vascular remodeling is a ubiquitous pathologic finding in patients with pulmonary hypertension (PH), a condition associated with impaired quality of life and decreased life expectancy 1. Despite treatment with potent vasodilating agents, pulmonary arterial hypertension (PAH) patients have a progressive disease course and extensive pulmonary vascular remodeling 1, 2. The development of more effective treatment options is hindered by our incomplete understanding of the cellular and molecular mechanisms of pulmonary vascular remodeling.

An unresolved question in PH is the identity of the parental cell type that generates the majority of cells found in remodeled pulmonary arteries (PAs). Earliest studies established the abundant and aberrant presence of cells with smooth muscle cell (SMC)‐like morphology as a main characteristic of pulmonary vascular remodeling 3, 4, 5, 6. Since then, the proposed repertoire of cell types that directly contribute to the neomuscularization has expanded to include major vascular resident cells from all three vessel layers, and bone marrow‐derived cells. The initial hypothesis of intermediate cells and pericytes as precursors of SMC‐like cells in neomuscularized PAs 7 was revisited using a lineage tracing approach 8. Additionally, endothelial cells (ECs), by endothelial‐to‐mesenchymal transition 9, and SMCs, through monoclonal expansion of specialized resident vascular SMCs 10, 11, were reported as potential sources of SMC‐like cells. Adventitial fibroblasts, either directly through transdifferentiation into myofibroblasts or indirectly by recruitment of circulating inflammatory cells, were also postulated as a contributing cell type to pulmonary vascular remodeling 12. One study using a lineage tracing approach did indeed indicate a structural contribution of bone marrow‐derived cells 13; however, follow‐up studies did not replicate these findings 14, 15. Finally, lung resident mesenchymal stem and progenitor cells were also described to structurally contribute to neomuscularization 16, 17.

Given these divergent findings, we performed a systematic study to assess the structural contribution of major resident vascular cell types to the remodeling process.

## Materials and methods

### Experimental animals

Animal experiments were approved by the Austrian Federal Ministry of Science, Research and Economy. Mice were maintained under specific pathogen‐free (SPF) conditions with controlled temperature and lighting, and allowed food and water *ad libitum*.

Mice carrying *CreERT2* under control of the smooth muscle actin [*Acta2‐CreERT2*; *Tg*(*Acta2‐cre/ERT2*)*12Pcn* 18] or smooth muscle myosin heavy chain promoter [*Myh11‐CreERT2*; *Tg*(*Myh11‐Cre/ERT2*)*1Soff* 19] were used to trace SMCs. Pericytes were traced using *CreERTM* under chondroitin sulfate proteoglycan 4 promoter [Jackson Laboratory, Bar Harbor, ME, USA; *Tg*(Cspg4‐cre/Esr1*)*BAkik* 20], fibroblasts using platelet‐derived growth factor receptor alpha‐driven *CreERT2* expression [*Tg*(*Pdgfra‐cre/ERT2*)*1Wdr* 21], and ECs with cadherin 5 promoter‐directed *CreERT2* expression [*Tg*(*Cdh5‐cre/ERT2*)*CIVE23Mlia* 22] transgenic mice. Induction of expression with tamoxifen, chronic hypoxia exposure, and house dust mite (HDM) treatment is provided in the supplementary material, Supplementary materials and methods.

Mouse lungs were flushed from blood with PBS, inflated with optimal tissue cutting compound (Sakura Finetek USA Inc, Torrance, CA, USA), fixed overnight in 1% paraformaldehyde at 4 °C followed by overnight incubation in 30% sucrose, and stored at −80 °C.

### Human lung tissue

Lung tissue from IPAH patients undergoing lung transplantation and downsized lung tissue from human donors were obtained from the Department of Surgery, Division of Thoracic Surgery, Medical University of Vienna, Austria, following written patient consent and approval by the institutional ethics committee (976/2010). Small resistance PAs (below the fourth branch of the main PA) were isolated from donors and IPAH patients, and stored in liquid nitrogen. Patient characteristics are given in the supplementary material, Table S1.

### Immunofluorescence and flow cytometry

Protocols for immunostaining of mouse lung cryosections or human formalin‐fixed, paraffin‐embedded lung sections and for flow cytometry are given in the supplementary material, Supplementary materials and methods. Antibody details are listed in the supplementary material, Table S2.

### Assessment of tdTomato incorporation in remodeled vessels

The incorporation of tdTomato‐labeled cells in remodeled vessels was assessed on lung tissue slides stained for αSMA and vWF. In the case of chronic hypoxia‐exposed mice, at least 20 randomly sampled intra‐acinary vessels below 35 μm in diameter per mouse were analyzed for the presence of tdTomato^+^ and αSMA^+^ cells. For HDM‐exposed mice, peribronchial and alveolar duct associated‐PAs were sampled and the number of tdTomato^+^ and αSMA^+^ cells counted. Only cells with visible nuclei were taken into consideration and a minimum of 30 αSMA^+^ cells in the case of normoxia/hypoxia, or 60 for saline/HDM, per slide were counted.

### Electron microscopy

Human lung samples were fixed in 2.5% glutaraldehyde and post‐fixed in osmium tetroxide. Following dehydration, samples were embedded in epoxy resin and cut to ultrathin sections, stained with uranyl acetate, and imaged using transmission electron microscopy (EM109, Zeiss, Oberkochen, Germany).

### Gene expression analysis

Details are provided in the supplementary material, Supplementary materials and methods. The primers used are listed in the supplementary material, Table S3.

### Statistics

Statistical analyses were performed in GraphPad Prism 5 using the Mann–Whitney or Welch's correction test. *P* values lower than 0.05 were considered statistically significant.

## Results

### Expansion of αSMA^+^ cells is a defining feature of pulmonary vascular remodeling in mice

The coverage of pulmonary vessels with αSMA^+^ cells was investigated on lung sections stained for vWF and αSMA in chronic hypoxia (hox) and allergen‐driven (house dust mite, HDM)‐induced pulmonary vascular remodeling (supplementary material, Figure S1). As these animal models have different underlying causative mechanisms, they partially address the heterogeneity in pathomechanism and progression commonly observed in PH patients. Chronic hypoxia induced a general shift towards more muscularization for all vessel sizes (supplementary material, Figure S1A, B). However, the strongest difference between normoxia and hypoxia was evident for vessels below 35 μm in diameter. These vessels were not fully muscularized under normoxic conditions, but became strikingly muscularized upon chronic hypoxia (supplementary material, Figure S1A, B). On the other hand, the HDM mouse model was manifested as significant thickening of more proximal pulmonary arteries 23 (supplementary material, Figure S1C, D).

**Figure 1:**
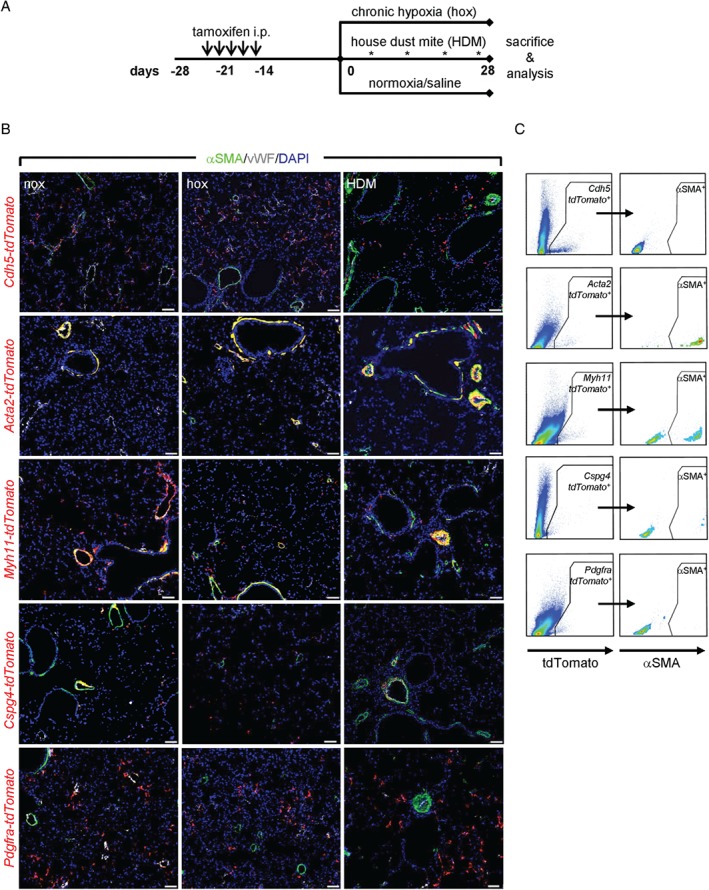
Assessment of pulmonary vascular remodeling in mice using a lineage tracing approach. (A) Schematic representation of lineage tracing of smooth muscle cells, pericytes, fibroblasts, and endothelial cells in pulmonary vascular remodeling models. (B) A low‐magnification overview portion of the lung slide co‐stained with alpha smooth muscle actin (αSMA, green), von Willebrand factor (vWF, white), and containing lineage‐labeled (red) endothelial cells (Cdh5‐tdTomato), smooth muscle cells (Acta2‐ and Myh11‐tdTomato), pericytes (Cspg4‐tdTomato), and fibroblasts (Pdgfra‐tdTomato) in control (nox), chronic hypoxia (hox), and house dust mite (HDM)‐exposed mouse lungs. Scale bar = 50 μm. (C) Representative dot blots of flow cytometric analysis for tdTomato and αSMA expression in control mouse lungs.

To address the structural contribution of major vascular cell types to the remodeling process, we applied a lineage tracing approach. Expression of fluorescent reporter tdTomato was induced by tamoxifen, which leads to permanent labeling of *CreERT2*‐expressing cells at the time, and all their subsequent progeny. Resident SMCs were labeled using *Acta2‐CreERT2*;*tdTomato* ^*flox*^ (*Acta2‐tdTomato* for conciseness) and *Myh11‐CreERT2*;*tdTomato* ^*flox*^ (*Myh11‐tdTomato*) mouse lines (supplementary material, Figure S1E). The contribution of pericytes to the expansion of αSMA^+^ vascular cells was assessed using the *Cspg4‐CreERTM*;*tdTomato* ^*flox*^ (*Cspg4‐tdTomato*) line, fibroblasts with the *Pdgfra‐CreERT2*; *tdTomato* ^*flox*^ (*Pdgfra‐tdTomato*) line, and ECs using *Cdh5‐CreERT2*;*tdTomato* ^*flox*^ (*Cdh5‐tdTomato*) mice (supplementary material, Figure S1E). As tamoxifen was given prior to hypoxia or HDM exposure (Figure 1A), the fate of pre‐existing labeled cells and their incorporation in remodeled vessels and co‐expression of αSMA were investigated.

### Lineage tracing of resident lung vascular cells

The endothelial mouse line (*Cdh5*‐*tdTomato*) showed incomplete labeling of ECs, in accordance with the original description in adult mice 22. Labeling of the interstitial capillary network was also apparent (Figure 1B); however, almost all *Cdh5‐tdTomato* ^+^ cells were αSMA^−^, as determined by flow cytometry (Figure 1C and supplementary material, Figure S2). The *Acta2‐tdTomato* mouse line labeled bronchial and vascular SMCs (Figure 1B and supplementary material, Figure S2) and constituted ∼80% of αSMA^+^ cells in both normoxic and hypoxic mouse lungs (Figure 1C and supplementary material, Figure S2). Similarly, *Myh11‐tdTomato* was used as an additional and independent SMC‐specific marker. The labeling pattern of vascular and bronchial SMCs using this line (Figure 1B) was similar to that observed with *Acta2‐tdTomato* mice, and *Myh11‐dTomato* cells constituted ∼50% of αSMA^+^ cells in normoxic and hypoxic mouse lungs (Figure 1C and supplementary material, Figure S2). However, we also observed additional labeling of parenchymal cells in *Myh11‐tdTomato* lungs (Figure 1B, C), most likely septal myofibroblasts adjoining the alveolar duct 24. The *Cspg4‐tdTomato* mouse line exhibited frequent labeling of a subpopulation of vascular SMCs in proximal PAs and αSMA^−^ cells in distal vessels (Figure 1B). In line with this, flow cytometry revealed that *Cspg4‐tdTomato* ^+^ cells represent ∼25% of αSMA^+^ cells in control and 40% in hypoxic lungs (Figure 1C and supplementary material, Figure S2). Of note, bronchial SMCs were *Cspg4‐tdTomato* ^−^. *Pdgfra‐tdTomato*‐labeled cells were detected mainly as single parenchyma‐embedded cells. Occasionally, these cells were present in the vicinity of both normal and remodeled vessels (Figure 1B). In control lungs, the majority of *Pdgfra‐tdTomato* ^+^ cells were negative for αSMA (Figure 1C and supplementary material, Figure S2).

### VEcad^+^ cells line the vessel lumen in normal and remodeled vessels

**Figure 2:**
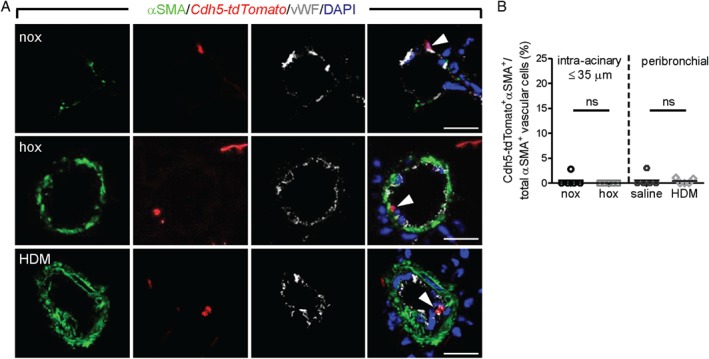
Assessment of endothelial cell contribution to pulmonary vascular remodeling. (A) Laser scanning confocal micrographs for the assessment of co‐localization of αSMA immunostaining with Cdh5‐tdTomato in pulmonary vessels. Arrowheads depict tdTomato‐vWF double‐positive cells. Scale bar = 20 μm. (B) Percentage of cells double‐positive for Cdh5‐tdTomato and αSMA label counted in at least 20 intra‐acinary vessels (≤ 35 μm; nox/hox) or 6–8 peribronchial and alveolar duct‐associated pulmonary arteries (saline/HDM) (n = 5 mice per group). A minimum of 30 (60) αSMA^+^ cells were counted per animal in nox/hox (saline/HDM) conditions. ns = not significant.

TdTomato^+^ cells lined the vessel lumen in both control and remodeled vessels, with no overlap with αSMA immunostaining in either model (Figure 2A). Furthermore, neither chronic hypoxia nor HDM exposure showed a significant contribution of *Cdh5‐tdTomato* ^+^ cells to αSMA^+^ cells (Figure 2B). The labeling efficiency of VEcad^+^ cells with tdTomato was ∼35% in all tested conditions (supplementary material, Figure S3A, B) without indication of tdTom‐VEcad‐αSMA triple‐positive cells (supplementary material, Figure S3C, D).

### SMCs are the major origin of αSMA^+^ cells found in remodeled vessels

**Figure 3:**
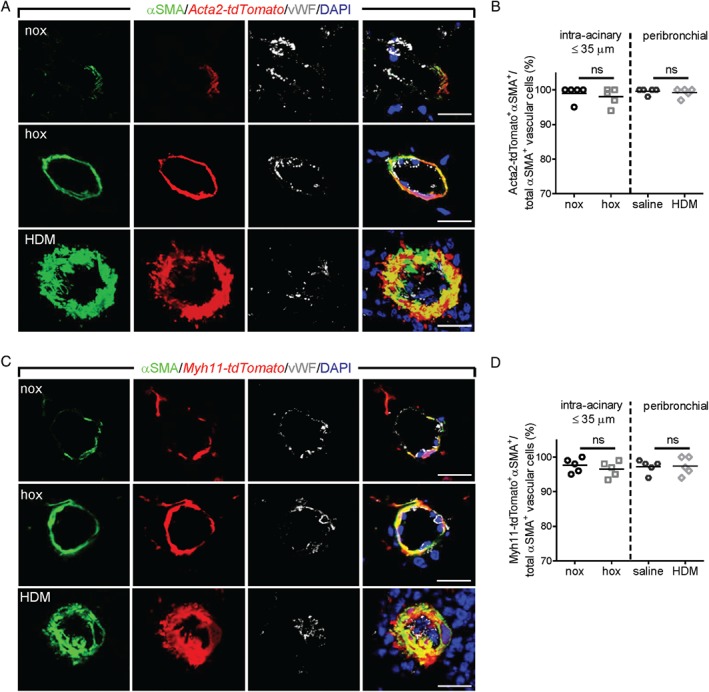
Assessment of the contribution of smooth muscle cells to pulmonary vascular remodeling. Laser scanning confocal micrographs for the assessment of co‐localization of αSMA immunostaining with Acta2‐tdTomato (A) and Myh11‐tdTomato (C) in pulmonary vessels. Scale bar = 20 μm. Percentage of cells double‐positive for αSMA and Acta2‐tdTomato (B) or Myh11‐tdTomato (D), counted in at least 20 intra‐acinary vessels (≤ 35 μm; nox/hox) or 6–8 peribronchial and alveolar duct‐associated pulmonary arteries (saline/HDM) (n = 5 mice per group). A minimum of 30 (60) αSMA^+^ cells were counted per animal in nox/hox (saline/HDM) conditions. ns = not significant.

In both *Acta2‐tdTomato* and *Myh11‐tdTomato* mice, we observed that almost all of the vascular tdTomato^+^ cells were αSMA^+^, evident as a significant co‐localization between the tdTomato signal and αSMA immunostaining (Figure 3A, C and supplementary material, Figure S4A–D). In line with this observation, more than 90% of αSMA^+^ cells were also tdTomato^+^, both in chronic hypoxia and in HDM‐remodeled vessels (Figure 3B, C). Of note, the *Myh11‐tdTomato* line exhibited additional labeling of SMMHC and αSMA‐immunonegative cells (supplementary material, Figure S4B–D).

### Neural glial antigen 2 (NG2/chondroitin sulfate proteoglycan 4)‐positive cells are found in normal and remodeled vessels

**Figure 4:**
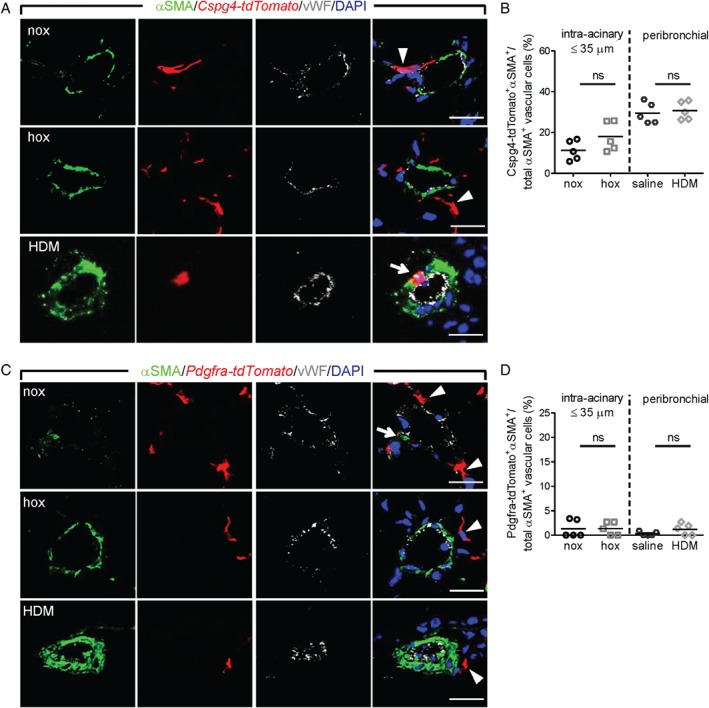
Assessment of the contribution of perivascular cells to pulmonary vascular remodeling. Laser scanning confocal micrographs for the assessment of co‐localization of αSMA immunostaining with Cspg4‐tdTomato (A) and Pdgfra‐tdTomato (C) in lung vessels. Arrows depict tdTomato‐αSMA double‐positive cells, while tdTomato single‐positive cells are depicted with arrowheads. Scale bar = 20 μm. Percentage of cells double‐positive for αSMA and Cspg4‐tdTomato (B) or Pdgfra‐tdTomato (D) label, counted in at least 20 intra‐acinary vessels (≤ 35 μm; nox/hox) or 6–8 peribronchial and alveolar duct‐associated pulmonary arteries (saline/HDM) (n = 5 mice per group). A minimum of 30 (60) αSMA^+^ cells were counted per animal in nox/hox (saline/HDM) conditions. ns = not significant.

*Cspg4‐tdTomato* ^+^ cells could already be detected in non‐muscularized parenchymal vessels (Figure 4A), while significant co‐localization between tdTomato and αSMA was observed mainly in a subset of cells in the medial layer from peribronchial and alveolar duct PAs (Figure 4A). The tdTomato labeling efficiency of NG2^+^ cells was between 50% and 80% (supplementary material, Figure S5A, B). The percentage of cells in vessels containing both *Cspg4‐tdTomato* ^+^ and αSMA^+^ markers increased slightly upon chronic hypoxia exposure, while in HDM‐exposed mice it remained constant, albeit at a higher percentage compared with intra‐acinary vessels (Figure 4B). However, *Cspg4‐tdTomato*‐αSMA‐NG2 triple‐positive cells represented a minor subpopulation of NG2‐immunoreactive perivascular and vascular cells (supplementary material, Figure S5C, D).

### Pdgfrα ^+^ cells are supporting perivascular cells lining the outer vessel wall

The majority of *Pdgfra‐tdTomato* ^+^ cells were present in lung parenchyma and represented ∼40% of cells immunolabeled with PDGFRα (supplementary material, Figure S6A, B). Confocal microscopy revealed that vessel‐associated *Pdgfra‐tdTomato* ^+^ cells were mainly located in perivascular regions and they displayed cytoplasmic projections that incorporated into vessel walls (Figure 4C). The majority of αSMA^+^ cells in both normal and remodeled vessels were negative for *Pdgfra‐tdTomato* (Figure 4C and supplementary material, Figure S6C, D), and *Pdgfra‐tdTomato* ^+^αSMA^+^ cells were observed only occasionally in HDM‐exposed mice (Figure 4D).

### Proliferation‐labeled SMCs from proximal PAs are found in distal neomuscularized vessels

**Figure 5:**
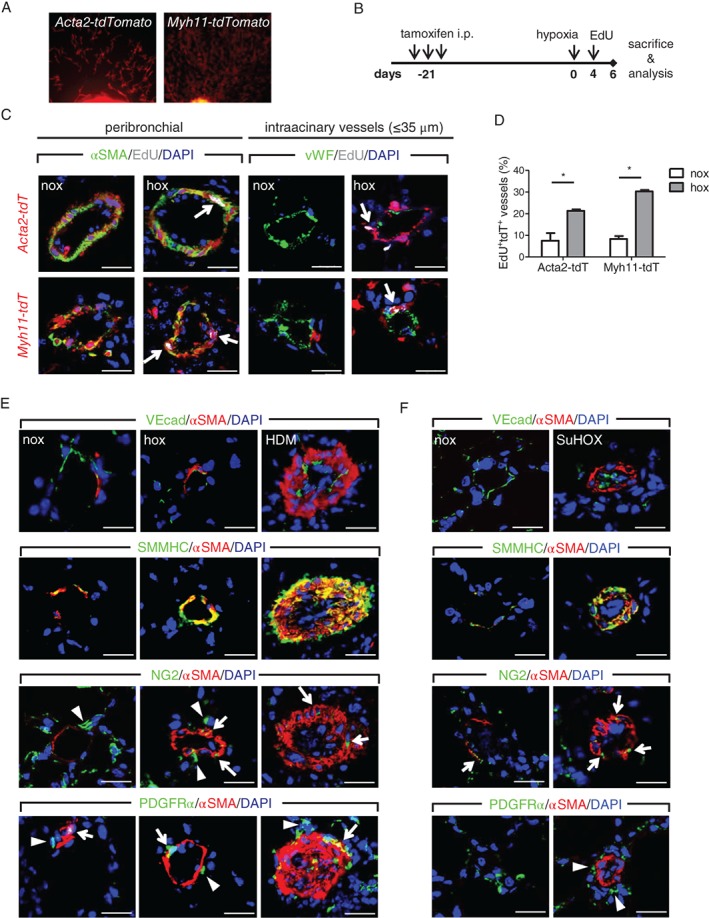
In vivo and ex vivo expansion of resident vascular smooth muscle cells. (A) Outgrowth of tdTomato‐labeled SMCs from main left mouse pulmonary artery tissue pieces. (B) Schematic representation to assess in vivo proliferation in a cell type‐specific manner. (C) Immunofluorescent images showing localization of proliferation label (EdU) in nuclei (white arrows) of either tdTomato‐αSMA double‐positive smooth muscle cells (SMCs) in peribronchial (left panels) or intra‐acinary vessels (right panels). Scale bar = 20 μm. (D) Percentage of pulmonary arteries present on the whole lung section containing tdTomato^+^αSMA^+^EdU^+^ positive cells (n = 2–3 mice per group for Acta2‐tdTomato; 3–4 for Myh11‐tdTomato). (E) Representative immunofluorescent co‐staining of alpha smooth muscle actin (αSMA), smooth muscle myosin heavy chain (SMMHC), vascular‐endothelial cadherin (VEcad), neural/glial antigen 2 (NG2), and platelet‐derived growth factor receptor alpha (PDGFRα) on control (nox), chronic hypoxia (hox), and house dust mite (HDM)‐exposed mouse lung samples. (F) Representative immunofluorescent co‐staining on rat control (nox) and Sugen5416‐hypoxia (SuHOX) lung samples. 4',6‐Diamidino‐2‐phenylindole (DAPI) was used as a nuclear counterstain. Arrows depict cells with overlapping αSMA and NG2/PDGFRα localization, while arrowheads mark cells with lack thereof. Scale bar = 20 μm.

Our lineage tracing approach identified SMCs as the main origin of αSMA^+^ cells that are found in remodeled arteries, both in chronic hypoxia and in the HDM model. This can either occur through the migration of SMCs from proximal to distal vessel regions or include proliferation of differentiated SMCs residing in proximal muscularized vessels. Recent research suggested that the proliferative and migratory capacity of resident SMCs was dependent on their anatomical position within the vessel wall 11. Here, we cultured tissue pieces dissected from the main, second, and third generation PA branches of *Acta2‐tdTomato* and *Myh11‐tdTomato* mice and observed migration and expansion of tdTomato^+^ SMCs (Figure 5A) and *in vitro* proliferative response to serum stimulation (supplementary material, Figure S7A, B). A combination of *in vivo* labeling of proliferating cells using EdU incorporation and SMC lineage tracing (Figure 5B) further showed that SMCs possess proliferative capacity irrespective of anatomical position and vessel size, evidenced by an increase in EdU^+^ tdTomato^+^ signals in αSMA‐immunoreactive cells in proximal, muscularized PAs from hypoxia‐exposed animals compared with controls (Figure 5C, D). Hypoxia‐induced proliferative response was not exclusive for SMCs, but was also evident in other resident cell types (supplementary material, Figure S7C–H).

### Localization of lineage markers is conserved between murine and human PAs

Next, we performed a comparative multicolor immunofluorescent staining of murine, rat, and human PAs. In murine and rat PAs, the endothelial marker VEcad localized to the thin luminal vessel layer and showed no overlap with αSMA (Figure 5E, F). Similar luminal localization and no overlap with αSMA were observed with additional endothelial markers CD31 and thrombomodulin (supplementary material, Figure S8A, B). This marker expression pattern was preserved in remodeled vessels from chronic hypoxia or HDM‐exposed mice and occluded vessels from SU5416/hypoxia‐exposed rats. SMC markers αSMA and SMMHC co‐localized to the medial vessel layer in controls and remodeled vessels in both mouse and rat models (Figure 5E, F). A fraction of NG2^+^ cells showed co‐expression of αSMA in control intra‐acinary vessels, while remodeled vessels in both murine and rat models displayed increased frequency in NG2–αSMA overlap (Figure 5E, F). The fibroblast marker PDGFRα was present in perivascular regions and showed rare overlap with αSMA (Figure 5E, F). PDGFRα^+^ cells in the αSMA^+^ layer were observed occasionally in HDM‐exposed mice (Figure 5E).

**Figure 6:**
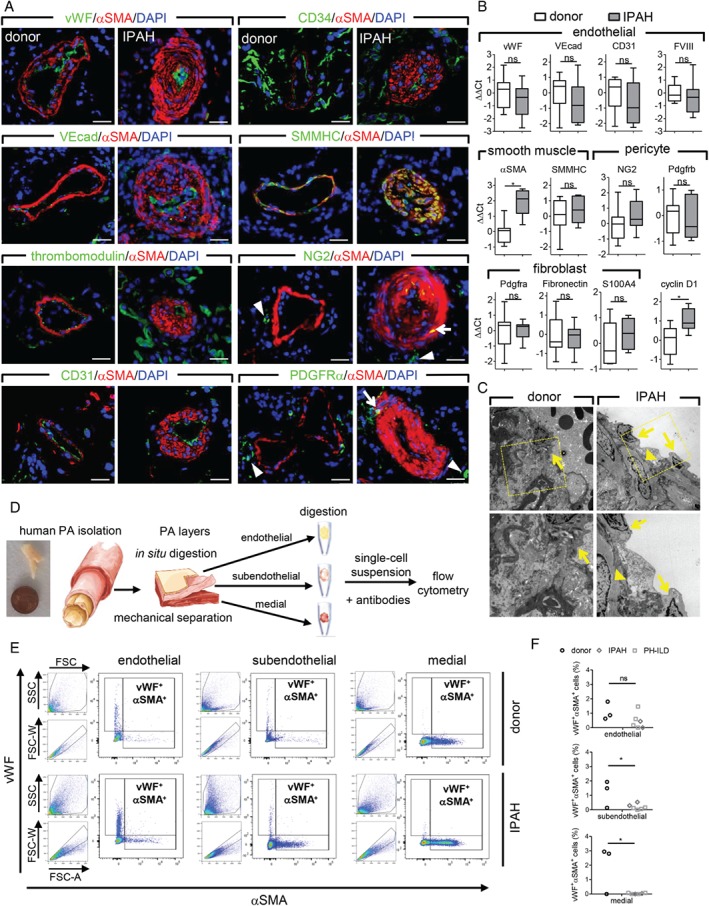
Localization and expression of lineage markers in human pulmonary arteries. (A) Representative immunofluorescent co‐staining of alpha smooth muscle actin (αSMA), von Willebrand factor (vWF), vascular‐endothelial cadherin (VEcad), thrombomodulin, CD31, CD34, smooth muscle myosin heavy chain (SMMHC), neural/glial antigen 2 (NG2), and platelet‐derived growth factor receptor alpha (PDGFRα) on donor (n = 4) and idiopathic pulmonary arterial hypertension (IPAH, n = 4) lung samples. Arrows depict cells with overlapping αSMA and NG2/PDGFRα localization, while arrowheads mark cells with lack thereof. 4',6‐Diamidino‐2‐phenylindole (DAPI) was used as a nuclear counterstain. Scale bar = 20 μm. (B) Quantitative PCR on isolated human pulmonary arteries (PAs) from donor (n = 12) and IPAH patients (n = 7). Gene expression of smooth muscle cell, endothelial, pericyte, fibroblast, and proliferation markers. Data are presented as a box‐and‐whisker plot depicting median with interquartile range (box) ± min/max (whiskers). (C) Representative electron microscopy images of an intimal region of PA. Arrows depict endothelial cells and arrowheads mark smooth muscle cells. (D) Schematic representation of the workflow for single cell analysis of isolated human PA layers. (E) Gating strategy and representative dot plots of flow cytometric analysis of vWF^+^αSMA^+^ cells in different PA layers from donor and IPAH. (F) Percentage of vWF^+^αSMA^+^ cells in PA layers from donors, IPAH, and PH due to interstitial lung disease (PH‐ILD) patients. *p < 0.05. ns = not significant.

We further compared the localization of the same lineage markers in PAs from donors and end‐stage IPAH patients (Figure 6A). The two SMC markers αSMA and SMMHC showed highly overlapping localization patterns and stained the medial layers of the vessel wall in both donor and IPAH PAs, as well as the neointimal layer in IPAH vessels (Figure 6A). Endothelial markers vWF, VEcad, thrombomodulin, CD31, and CD34 displayed a staining pattern limited to a single‐cell layer lining the luminal side of both donor and IPAH PAs (Figure 6A). In addition, VEcad, thrombomodulin, CD31 and CD34 displayed a broader staining pattern in the lung parenchyma, depicting the capillary bed, inflammatory cells or adventitial regions (Figure 6A). The pericyte marker NG2 was predominantly located in single, isolated perivascular cells and showed a weaker, variable intensity in αSMA‐stained regions (Figure 6A). The fibroblast marker PDGFRα stained the outermost vessel layer, and this localization was consistent with that of adventitial fibroblasts (Figure 6A). The only notable difference was observed in the case of plexiform lesions, in which endothelial markers stained the majority of cells in the lesion forming multiple channel‐like layers supported by αSMA^+^ cells or complete occlusion surrounded by SMC marker‐expressing cells (supplementary material, Figure S9). We have substantiated the examination of lineage markers by gene expression analysis from human resistance pulmonary arteries (below fourth branch) isolated from donor and IPAH patients. The expression levels of αSMA and proliferation marker cyclin D1 were significantly higher in PAs from IPAH patients than in those from donors (Figure 6B). In contrast, the expression levels of endothelial, pericyte, and fibroblast markers were not changed in IPAH compared with donor PAs (Figure 6B).

The close proximity of SMC and EC markers makes assessment of their possible co‐localization using standard immunofluorescent staining of tissue slides challenging. We therefore evaluated the ultrastructural composition of IPAH PAs using electron microscopy (Figure 6C). In normal PAs, a single‐cell layer of endothelium was observed, followed directly by a connective tissue region (Figure 6C). In IPAH pulmonary arteries with neointima, ECs were present as a single‐cell luminal layer, with SMCs located adjacent to and in direct contact with the EC layer. In all investigated cases, ECs and SMCs showed clearly differentiated and distinctive morphologies, without any indication for a transition phenotype between the two cell types. Additionally, we refined a PA digestion protocol that allowed the quantification of vascular cells expressing both EC and SMC markers on a single cell level (Figure 6D). Flow cytometric analysis of single cell suspensions from isolated PA layers (endothelial, subendothelial, and medial) showed a negligible number of vWF^+^αSMA^+^ cells and no discernible difference between donors and PH patients (ranging from 0 to 17 out of 116 to 6400 gated events; Figure 6E, F).

## Discussion

Pulmonary vascular remodeling in IPAH patients is a hallmark of disease pathology, has a variable progression, and is mostly unresponsive to current therapies 2. Understanding the origin of cells that make up the remodeled pulmonary vessel wall in IPAH has so far been elusive. Current lineage tracing approaches were based on only one model and reported conflicting results regarding the contribution of different cell types 8, 9, 10, 11, 13, 17. In this study, we investigated the origin of SMC‐like cells in remodeled PAs using two different murine models and encompassing all major vascular cell types. This was complemented with systematic assessment of cell type‐specific marker expression and localization in human PAs. The major findings of our study are (1) that resident SMCs are a major source of αSMA^+^ cells that appear in the remodeled PAs, and (2) that IPAH patients and murine models of pulmonary vascular remodeling show overall similar expression and localization patterns for various vascular cell‐type markers.

The earliest studies addressing the cellular composition and changes were based on ultrastructural analysis and *in vivo* labeling of proliferating cells from chronic hypoxia and monocrotaline‐exposed rats, and postulated that new smooth muscle cells differentiate from precursor smooth muscle cells – the pericyte and intermediate cell 4, 6, 25. As cell type‐specific and inducible *Cre* recombinase‐expressing rats are not available, validation of these observations is limited to murine models. The earliest report using lineage tracing to investigate pulmonary vascular remodeling reported the contribution of bone marrow‐derived cells 13. However, the reported frequency of bone marrow‐derived cells associated with remodeled vessels was too low to account for the bulk of cells present in these vessels, while co‐localization with SMC markers could not be reproduced 15, 16.

Thus, we focused our efforts on major lung resident cell types in chronic hypoxia and HDM models that display different aspects of pulmonary vascular remodeling. Chronic hypoxia is associated with remodeling of the intra‐acinar artery bed, since the majority of neomuscularized vessels express the ephrin‐b2 arterial marker 26. On the other hand, HDM‐induced remodeling is characterized by striking thickening of peribronchial PAs 23, which resemble those observed in human samples. Further similarity was the matching localization of cell type‐specific markers in normal and remodeled PAs. Pulmonary arteries in human, mouse, and rat have a single luminal cell layer characterized by expression of endothelial markers (vWF, VEcad, thrombomodulin, CD31), followed by an αSMA^+^SMMHC^+^ layer and a PDGFRα^+^ adventitial layer. This shared pattern of marker expression between human and rodents could indicate an evolutionary conserved mechanism for pathologic pulmonary vascular remodeling between species.

Going beyond previous studies 10, 11, we showed that the expansion of resident SMCs, not only in the hypoxia but also in the HDM model, is the major parental cell type responsible for PA remodeling. Constantly high overlap between tdTomato reporter and αSMA immunostaining clearly demonstrates that the vast majority of αSMA^+^ cells present in neomuscularized vessels in the hypoxia model and thickened PAs in the HDM model are progeny of SMCs. We further show that the partial coverage of normal murine parenchymal vessels with αSMA^+^ cells extends throughout the pulmonary vascular bed, without a clearly defined muscularization border, in contrast to the reported existence of a muscularization border 10, 11. Whereas we applied an unbiased random sampling of pulmonary vessels, previous studies focused on a single supernumerary branch PA near to a bronchus. Such supernumerary branches very often show an abrupt lack of αSMA^+^ cells, which is not representative of a graded transition from circular full coverage to partial, mesh‐like coverage of PAs with αSMA^+^ cells 27.

Using *in vivo* EdU‐labeling and *ex vivo* experiments with pulmonary artery tissue explants, we provide evidence supporting the notion that proliferation of resident vascular SMCs is the main pathomechanism of pulmonary vascular remodeling. Cumulatively, these results indicate that plasticity and proliferative capacity might be a characteristic of all SMCs in the pulmonary artery tree, irrespective of vessel caliber and anatomical position. Even though hypoxia exposure increased proliferation of other resident cell types (our observation and refs 4, 28), we found no evidence that these cells acquired αSMA expression. A previous study reported that the expansion of a specialized subpopulation of SMCs is accompanied by the transient loss of SMMHC expression during the vascular remodeling process 11. It is likely that with the inclusion of more markers in future studies, such as desmin and meta‐vinculin, a more complex picture of multiple SMC subpopulations in human disease might emerge, similar to observations made in animal models 29.

Consistent with the data from murine models, we observed increased expression of the αSMA cell type marker and proliferation marker cyclin D1 in isolated human PAs from IPAH patients, corroborating previous observations of increased percentages of SMCs positive for PCNA or Ki‐67 proliferation marker in PAs from IPAH patients 30, 31. However, direct proof of lineage origin of neointimal cells in IPAH patients is still missing. Furthermore, it remains to be determined whether the increased SMC proliferative response in human disease is a consequence of SMC intrinsic changes and autocrine factors or rather perturbed paracrine and endocrine signaling. A significant drawback in better understanding and deciphering of underlying mechanisms is the lack of a murine model that fully recapitulates the remodeling seen in IPAH patients. This model could potentially lead to the development of novel therapies that would address the extensive and severe neointima formation and collagen deposition that is still seen in end‐stage IPAH patients treated with modern vasodilative therapy 32.

Our results showed two major subpopulations of NG2^+^ cells, a marker commonly used to identify pericytes. One population that co‐expresses αSMA even under normal conditions is observed in partially muscularized intra‐acinary vessels and more frequently in peribronchial PAs. A second population of NG2^+^ cells that is mostly negative for αSMA was observed frequently as perivascular cells. Whether the *Cspg4‐tdTomato* ^*+*^ cells incorporated in remodeled vessels, also observed in a previous study 8, come from the αSMA^+^ or αSMA^−^ subpopulation of NG2‐expressing cells would have to be addressed in further lineage tracing studies. Generally, the classification and functional behavior of pericytes are still uncertain. It is possible that NG2 is expressed by different cell types 16 or marks subpopulations with alternative roles in pulmonary vascular remodeling. Studies using the alternative pericyte marker 3G5 showed a functional role of these cells in remodeling by supporting proper vessel coverage through interaction with the endothelium 33, 34. Taken together, it seems likely that pericytes are not a major source of SMC‐like cells in neomuscularized vessels.

The increased coverage with Pdgfrα‐immunoreactive cells was evident only in the chronic hypoxia model, but lineage‐labeled cells from the *Pdgfra‐tdTomato* mouse line did not contribute to the αSMA^+^ vascular cell pool in remodeled vessels, except for their rare presence in HDM‐remodeled vessels. However, unlike the frequent presence of *Pdgfra‐tdTomato* ^+^ cells in the parenchyma, perivascular localization of these cells was generally a sporadic event. Thus, we cannot exclude a possibility of *de novo* expression of Pdgfrα from an alternative cell population. Nevertheless, these results are in line with a strong proliferative response of adventitial fibroblasts upon hypoxia 35. The validity of PDGFRα as an adventitial fibroblast marker was further confirmed on human samples but, similar to the murine models, showed no significant overlap with SMC markers. Consistent with these results are reports from IPAH and PH patients with associated lung diseases showing only limited adventitial thickening 2, 32, 36. Therefore, the structural contribution of adventitial fibroblast‐derived SMC‐like cells to pulmonary vascular remodeling may represent a very rare event.

In our final set of lineage tracing experiments, we used the *Cdh5‐tdTomato* mouse line to investigate the potential contribution of endothelial‐to‐mesenchymal transition (EndoMT) to pulmonary vascular remodeling. In contrast to other endothelial *CreERT2* mouse lines such as *Tie2*, *Cdh5‐tdTomato* does not show labeling of bone marrow‐derived cells 22. A lineage tracing study using *Cdh5‐tdTomato* on a monocrotaline pyrrole/pneumectomy model showed limited overlap between lineage‐traced ECs and SMC markers 9. In PAH and rat Sugen/hypoxia model, EndoMT was estimated to occur in 5% of vessels, based on co‐expression of SMC and endothelial markers 37. Similarly, co‐localization of VE‐cadherin and CD31 with αSMA was reported in intimal and plexiform lesions 38. In contrast, another study investigating cell type marker localization and expression in human plexiform lesions and adjacent PAs found no evidence of co‐expression of SMC and EC markers in either plexiform or concentric lesions 30. Furthermore, gene expression analysis of laser‐microdissected PAs from controls and PAH patients showed an increase in αSMA but no change in CD31 endothelial marker expression 30. Similarly, we also observed increased expression of αSMA but not of any tested endothelial markers in isolated PAs from IPAH patients compared with donors. We also found no presence of cells in remodeled PAs that co‐express the SMC marker αSMA and EC markers. Close proximity of SMCs and adjacent ECs, fixation and staining artefacts, and limiting imaging resolution could possibly explain the observed discrepancies. An additional difficulty is the comparison of lineage markers that are expressed in different cellular compartments. The majority of endothelial markers are most abundant on the cell surface (VEcad, CD31, thrombomodulin, CD34), while the most commonly used markers for differentiated SMCs (αSMA, SMMHC) are intracellular. This would make the assessment of co‐localization between a cell surface and intracellular markers technically challenging on tissue slides, particularly without clearly visible cell borders and nuclei. Therefore, we included vWF as an additional EC marker that is normally present in intracellular vesicles, allowing an easier assessment of co‐localization with other intracellular markers. Importantly, remodeled human, mouse, and rat PA each displayed increased thickness of a distinct αSMA^+^SMMHC^+^ vessel layer that was clearly distinguishable from the single‐cell luminal layer immunoreactive for endothelial markers. Even the plexiform lesions retained the distinct pattern of separate SMC‐ and EC‐marker localization. Ultrastructure analysis of IPAH PA further verified that cells having characteristic SMC morphology are present in the neointima and in direct contact with the ECs lining the lumen. A similar observation of SMCs subadjacent to ECs has been made in the rat chronic hypoxia model 7. These results were further confirmed using freshly isolated human PA and flow cytometry of αSMA and vWF co‐expression, allowing unbiased quantification of SMC–EC marker co‐expression on a single cell level. PH patients (IPAH and ILD‐PH) showed no increase of vWF^+^αSMA^+^ cell counts compared with donor. This double‐positive cell population represented a minor percentage (generally below 1%) of total gated events in both donors and PH patients. A potential limiting factor in this analysis was a low absolute number of cells that can be isolated from small PAs and the possibility that some gated events might represent false positives due to residual cell debris/dead cells.

However, due to limitations of the current murine models, it is still not possible to draw definitive conclusions about the presence and extent of EndoMT in the human disease. Several EC phenotypical changes (e.g. cuboidal appearance, protrusions into the lumen, hypertrophy, cell surface marker expression, vWF release) could be a sign of EC activation 39, 40 rather than evidence of permanent and complete transdifferentiation. While ECs have a clear functional role in directing the remodeling process by the release of paracrine factors such as PDGF‐BB 11, their potential contribution to neointima formation requires further investigation.

## Author contributions statement

SC, LMM, and GK conceived the study and drafted the manuscript. SC, LMM, ESP, and WB carried out experiments and performed data analysis. BG and WK performed data collection. EEA, RV, HO, SB, and AO contributed to study design and data interpretation. All the authors had final approval of the submitted manuscript.

**Abbreviations**
αSMA: alpha smooth muscle actin
ECs: endothelial cells
HDM: house dust mite
hox: hypoxia
(I)P(A)H: (idiopathic) pulmonary (arterial) hypertension
NG2: neural glial antigen 2 (chondroitin sulfate proteoglycan 4)
nox: normoxia
PA: pulmonary artery
PDGFRα: platelet‐derived growth factor receptor alpha
SMCs: smooth muscle cells
SMMHC: smooth muscle myosin heavy chain
VEcad: vascular endothelial cadherin

**SUPPLEMENTARY MATERIAL ONLINE**

**Supplementary materials and methods**

**Figure S1.** Assessment of pulmonary vascular remodeling in mice

**Figure S2.** Flow cytometric analysis of lineage labeling

**Figure S3.** Labeling efficiency of *Cdh5‐tdTomato* mouse line

**Figure S4.** Labeling efficiency of *Myh11‐tdTomato* mouse line

**Figure S5.** Labeling efficiency of *Cspg4‐tdTomato* mouse line

**Figure S6.** Labeling efficiency of *Pdgfra‐tdTomato* mouse line

**Figure S7.** Proliferative capacity of (peri)vascular resident cells

**Figure S8.** Localization of lineage markers in rat pulmonary arteries

**Figure S9.** Localization of lineage markers in plexiform lesions from IPAH patients

**Table S1.** Patient characteristics

**Table S2.** Antibody details

**Table S3.** Primer sequences

## Supporting information


**Supplementary materials and methods**

**Figure S1. Assessment of pulmonary vascular remodeling in mice.** (A) Ratio muscularization (αSMA‐positive proportion of vessel wall circumference), control (nox), n = 1281; chronic hypoxia (hox)‐exposed mice, n = 1351 vessels. Majority of vessels in controls with diameter < 35 μm are not fully muscularized (gray region) . (B) Distribution of vessels according to ratio muscularization. (C) Medial wall thickness of peribronchial/alveolar duct arteries (n = 441 control, n = 449 HDM‐exposed mice). Majority of arteries in controls have wall thickness bellow 20%. (D) Distribution of arteries according to medial wall thickness. Inserts (B;D) show representative double IHC staining of vessels against αSMA (purple) and von Willebrand factor (brown). Scale bar = 10 μm. (E) Generation of cell‐type specific conditional tdTomato reporter transgenic mice under αSMA (Acta2‐CreERT2), smooth muscle myosin heavy chain (Mhy11‐CreERT2), neural/glial antigen 2 (Cspg4‐CreERTM), platelet‐derived growth factor receptor alpha (Pdgfra‐CreERT2), and vascular‐endothelial cadherin (Cdh5‐CreERT2) promoter control.
**Figure S2. Flow cytometric analysis of lineage labeling.** Percentage of tdTomato^+^αSMA^+^ cells within total tdTomato+ cells from lungs of normoxia and chronic hypoxia‐exposed mice (n = 2‐5 mice/group). Each point represents a single animal and with line depicting mean value.
**Figure S3. Labeling efficiency of Cdh5‐tdTomato mouse line.** (A) Representative laser scanning confocal micrographs for the assessment of co‐localization of VEcad and αSMA‐immunostaining with Cdh5‐tdTomato. Arrows depict tdTomato‐VEcad double positive cells, while tdTomato single positive cells are depicted with arrowheads. White scale bar depicts 20 μm. (B) Quantification of tdTomato labeling efficiency. (C) Percentage of VEcad+ cells co‐labeled with tdTomato and αSMA. (D) Percentage of Cdh5‐tdTomato+ cells co‐labeled with VEcad and αSMA. Each point represents a measurement based on at least 60 VEcad+ cells in a single animal.
**Figure S4. Labeling efficiency of Myh11‐tdTomato mouse line.** (A) Representative laser scanning confocal micrographs for the assessment of co‐localization of SMMHC and αSMA‐immunostaining with Myh11‐tdTomato. Arrows depict tdTomato‐SMMHC double positive cells, while tdTomato single positive cells are depicted with arrowheads. White scale bar depicts 20 μm. (B) Quantification of tdTomato labeling efficiency. (C) Percentage of SMMHC+ cells co‐labeled with tdTomato and αSMA. (D) Percentage of Myh11‐tdTomato+ cells co‐labeled with SMMHC and αSMA. Each point represents a measurement based on at least 40 SMMHC+ cells in a single animal.
**Figure S5. Labeling efficiency of Cspg4‐tdTomato mouse line.** (A) Representative laser scanning confocal micrographs for the assessment of co‐localization of NG2 and αSMA‐immunostaining with Cspg4‐tdTomato. Arrows depict tdTomato‐NG2 double positive cells, while tdTomato single positive cells are depicted with arrowheads. White scale bar depicts 20 μm. (B) Quantification of tdTomato labeling efficiency. (C) Percentage of NG2+ cells co‐labeled with tdTomato and αSMA. (D) Percentage of Cspg4‐tdTomato+ cells co‐labeled with NG2 and αSMA. Each point represents a measurement based on at least 110 NG2+ cells in a single animal.
**Figure S6. Labeling efficiency of Pdgfra‐tdTomato mouse line.** (A) Representative laser scanning confocal micrographs for the assessment of co‐localization of PDGFRaand αSMA‐immunostaining with Pdgfra‐tdTomato. Arrows depict tdTomato‐PDGFRa double positive cells, while tdTomato single positive cells are depicted with arrowheads. White scale bar depicts 20 μm. (B) Quantification of tdTomato labeling efficiency. (C) Percentage of PDGFRa+ cells co‐labeled with tdTomato and αSMA. (D) Percentage of Pdgfra‐tdTomato+ cells co‐labeled with Pdgfraand αSMA. Each point represents a measurement based on at least 75 Pdgfra+ cells in a single animal.
**Figure S7. Proliferative capacity of (peri)vascular resident cells.** (A) Acta2‐tdTomato+ or (B) Myh11‐tdTomato+ cells were isolated from the main left pulmonary artery tissue pieces (n = 4 mice) and their in vitro proliferative response to 10% fetal calf serum (FCS) measured using thymidine incorporation assay. (C‐E) Representative images showing localization of proliferation label (EdU) in nuclei (white arrows) with VEcad, NG2 and PDGFRa immunostaining in peribronchial arteries from Acta2‐tdTomato (C,E) or Cspg4‐tdTomato reporter mice. White scale bar depicts 20 μm. Percentage of VEcad‐ (F), NG2‐ (H), and PDGFRa‐ (H) positive (peri)vascular cells labeled with EdU (n = 2‐3 mice/group, n = 55‐135 nuclei/mouse).
**Figure S8. Localization of lineage markers in rat pulmonary arteries.** Representative immunofluorescent co‐staining of alpha smooth muscle actin (αSMA), CD31, thrombomodulin, and von Willebrand factor (vWF) on (A) mouse (normoxia/saline‐nox, chronic hypoxia‐hox, house dust mite‐HDM) and (B) rat (nox, SU5416/hypoxia) lung samples. 4',6‐diamidino‐2‐phenylindole (DAPI) was used as nuclear counterstain. White scale bar depicts 20 μm.
**Figure S9. Localization of lineage markers in plexiform lesions from IPAH patients.** Representative immunofluorescent co‐staining of alpha smooth muscle actin (αSMA), von Willebrand factor (vWF), thrombomodulin, CD31, and CD34 on plexiform lesions. 4',6‐diamidino‐2‐phenylindole (DAPI) was used as nuclear counterstain. White scale bar depicts 50 μm.

**Table S1.** Patient characteristics
**Table S2.** Antibody details
**Table S3.** Primer sequences
