# Supplementary material for: Resident cell lineages are preserved in pulmonary vascular remodeling
Source: J Pathol. 2018 Mar 9;244(4):485–98. doi: 10.1002/path.5044 (PMC5903372; doi:10.1002/path.5044)
Supplement: Supplementary file 1 — Supplementary materials and methods [file PATH-244-485-s001.docx]

**Supplementary materials and methods**

Reference numbers refer to the main text reference list

*Experimental animals*

TdTomato expression was induced in 5- to 7-week-old mice by three to five consecutive single daily intraperitoneal (i.p.) injections of tamoxifen (Sigma, Vienna, Austria) at a dose of 3 mg per animal. Mice carrying *Cdh5-CreERT2* transgene received an additional tamoxifen injection (1 mg per mouse) at postnatal day 10. After a resting period of at least 2 weeks, mice were either exposed to 10% normobaric chronic hypoxia, as described previously [1], or were given an intranasal (i.n.) application of 50 µg of house dust mite allergen (Greer Labs, Lenoir, NC, USA) once per week for a total of 4 weeks. Development of PH in chronic hypoxia-exposed mice was confirmed using invasive right ventricular pressure measurement and by determination of Fulton index as described previously [26]. Successfulness of HDM allergen challenge was confirmed by lung function measurements (Flexivent, Scireq, Montreal, Canada). *In vivo* labeling of proliferating cells was done using a single i.p. injection of the thymidine analog EdU (5-ethynyl-2'-deoxyuridine, 50 mg/kg; Thermo Fisher Scientific, Waltham, MA, USA). Animal numbers are given in the figure legends.

Main, secondary, and tertiary pulmonary arteries from the left lung lobe were isolated from *Acta2-CreERT2* and *Myh11-CreERT; tdTomato^flox^*. PA tissue pieces were either snap-frozen and stored in liquid nitrogen or plated in growth medium containing 20% fetal bovine serum for cell outgrowth and expansion, followed by sorting and further *in vitro* subculturing of tdTomato^+^ cells.

For the rat experiments, male Sprague Dawley rats (weighing approximately 250 g) were given an i.p. injection of SU5416 (20 mg/kg) and exposed for 4 weeks to hypoxia (FiO_2_ = 10%), followed by 2 weeks of normoxia.

*Immunofluorescence*

Five-micrometer cryosections were fixed and permeabilized with ice-cold methanol/acetone (1:1), washed with PBS, and blocked in 5% donkey serum (Jackson Immuno Research Laboratories, West Grove, PA, USA). Human formalin –fixed, paraffin-embedded lung sections were dewaxed and subjected to heat-induced antigen retrieval at pH 6, followed by 5% donkey serum block. Primary antibodies (listed in the supplementary material, Table S2) were applied overnight at 4°C in 0.1% BSA. After washing steps, sections were incubated with donkey anti-rabbit, anti-goat or anti-mouse immunoglobulin labeled with Alexa Fluor dyes 488, 555 or 647 (all from Thermo Fisher Scientific, 1:500). The tyramide amplification kit was used for the detection of PDGFRα and NG2 (Thermo Fisher Scientific) in combination with HRP-labelled anti-rabbit antibody (Thermo Fisher Scientific) or ImmPress Reagent (Vector Laboratories, Burlingame, CA, USA). Nuclear counterstaining was performed with DAPI mounting medium (Vector Laboratories). Incorporation of EdU was detected using a Click-iT Assay Kit (Thermo Fisher Scientific).

*Assessment of tdTomato induction efficiency and pulmonary vascular remodeling*

Efficiency of tamoxifen induction was analyzed on cryosections from 3–4 animals per group that were co-stained with cell-type markers anti-VEcad, -SMMHC, -NG2, or -PDGFRα and anti-αSMA. A minimum of 130 cells positive for the respective cell-type marker was counted for each treatment group and results were expressed as a percentage of tdTomato^+^ cells to the total number of cell-type marker^+^ cells. Additionally, the percentage of triple-positive cells (tdTomato^+^cell-type marker^+^αSMA^+^) in a population of tdTomato^+^ or cell-type marker^+^ cells was calculated.

The ratio of muscularization and vessel wall thickness was analyzed on historic in-house formalin-fixed, paraffin-embedded tissue slides from chronic hypoxia and HDM-exposed mice. Double immunohistochemical staining against αSMA and vWF and semi-automated remodeling analysis were carried out as previously described [41].

*Flow cytometry*

Mouse lungs were flushed with PBS, inflated with dispase (Corning, Bedford, MA, USA), and digested for 1 h at 37°C. Tissues were then cut and cells collected in medium containing DNase I (Roche, Mannheim, Germany). Cell suspensions were fixed on ice with 1% paraformaldehyde (PFA), washed, incubated with 0.3% saponin, stained with FITC-labelled anti-αSMA, and analyzed by flow cytometry. Gating was set to exclude doublets and cells measured for tdTomato and SMA expression. Negative controls included unstained and tdTomato-negative cells, samples stained with a single color (tdTomato or SMA). TdTomato-αSMA double-positive cells from *Acta2-tdTomato* mice were used as a positive control. Initial gating was based on FSC and SSC physical characteristics to remove debris, doublets, and the majority of dead cells. Autofluorescence was determined in the negative controls. Compensations were made using single stained samples, while unlabeled and single stained samples served to set negative gates.

Small resistance human PAs were isolated from donors, IPAH or PH due to interstitial lung disease (ILD-PH) patients. The endothelial layer was removed by gentle scraping after *in situ* enzymatic digestion (mixture of collagenase I, dispase, and DNase I), followed by careful mechanical separation of the intimal and medial layers. Samples were processed with collagenase I and DNaseI, fixed with PFA, incubated with 0.3% saponin, stained with anti-αSMA (FITC) and anti-vWF (PE), and measured by flow cytometry. Negative controls included unstained cells, samples stained with a single color (vWF or SMA). Initial gating was based on FSC and SSC physical characteristics to remove debris, doublets, and the majority of dead cells. Autofluorescence was determined in the negative controls. Compensations were made using single stained samples, while unlabeled and single stained samples served to set negative gates.

*Gene expression analysis*

RNA was isolated from human PA tissues (RNeasy Micro Kit; Qiagen, Hilden, Germany), amplified (Ovation PicoSL WTA System V2 kit; NuGen Technologies, San Carlos, CA, USA), and gene expression of selected markers was performed (QantiFast SYBR, Qiagen). *PBGD* and *B2M* served as reference genes. The sequence of the applied primers is given in the supplementary material, Table S3. Melting curve analysis and gel electrophoresis were performed to confirm the exclusive amplification of the expected PCR product. The ΔCt values for each target gene were calculated as ΔCt = Ct reference gene – Ct target gene. The ΔΔCt values were calculated as ΔΔCt = ΔCt sample – mean ΔCt of control group.

*Proliferation assay*

Proliferation of Acta2-tdTomato^+^ and Mhy11-tdTomato^+^ cells sorted from mouse PAs was assessed using a thymidine incorporation assay. Briefly, cells plated in a 96-well plate were serum-starved overnight. The following day, cells were stimulated with fetal bovine serum (10%) for 24 h with simultaneous addition of [^3^H]thymidine (Biotrend Chemikalien, Cologne, Germany). The amount of radioactivity incorporated in DNA was measured by a scintillation counter (1450 Microbeta Trilux liquid scintillation and luminescence counter; Wallac, Perkin Elmer, Monza, Italy).
