# Supplementary material for: Resident cell lineages are preserved in pulmonary vascular remodeling
Source: J Pathol. 2018 Mar 9;244(4):485–98. doi: 10.1002/path.5044 (PMC5903372; doi:10.1002/path.5044)
Supplement: Supplementary file 2 — Figure S1. Assessment of pulmonary vascular remodeling in mice. (A) Ratio muscularization (αSMA‐positive proportion of vessel wall circumference), control (nox), n = 1281; chronic hypoxia (hox)‐exposed mice, n = 1351 vessels. Majority of vessels in controls with diameter < 35 μm are not fully muscularized (gray region) . (B) Distribution of vessels according to ratio muscularization. (C) Medial wall thickness of peribronchial/alveolar duct arteries (n = 441 control, n = 449 HDM‐exposed mice). Majority of arteries in controls have wall thickness bellow 20%. (D) Distribution of arteries according to medial wall thickness. Inserts (B;D) show representative double IHC staining of vessels against αSMA (purple) and von Willebrand factor (brown). Scale bar = 10 μm. (E) Generation of cell‐type specific conditional tdTomato reporter transgenic mice under αSMA (Acta2‐CreERT2), smooth muscle myosin heavy chain (Mhy11‐CreERT2), neural/glial antigen 2 (Cspg4‐CreERTM), platelet‐derived growth factor receptor alpha (Pdgfra‐CreERT2), and vascular‐endothelial cadherin (Cdh5‐CreERT2) promoter control. Figure S2. Flow cytometric analysis of lineage labeling. Percentage of tdTomato+αSMA+ cells within total tdTomato+ cells from lungs of normoxia and chronic hypoxia‐exposed mice (n = 2‐5 mice/group). Each point represents a single animal and with line depicting mean value. Figure S3. Labeling efficiency of Cdh5‐tdTomato mouse line. (A) Representative laser scanning confocal micrographs for the assessment of co‐localization of VEcad and αSMA‐immunostaining with Cdh5‐tdTomato. Arrows depict tdTomato‐VEcad double positive cells, while tdTomato single positive cells are depicted with arrowheads. White scale bar depicts 20 μm. (B) Quantification of tdTomato labeling efficiency. (C) Percentage of VEcad+ cells co‐labeled with tdTomato and αSMA. (D) Percentage of Cdh5‐tdTomato+ cells co‐labeled with VEcad and αSMA. Each point represents a measurement based on at least 60 VEcad+ cells in a single [file PATH-244-485-s002.pdf]

Supplementary Figure S1

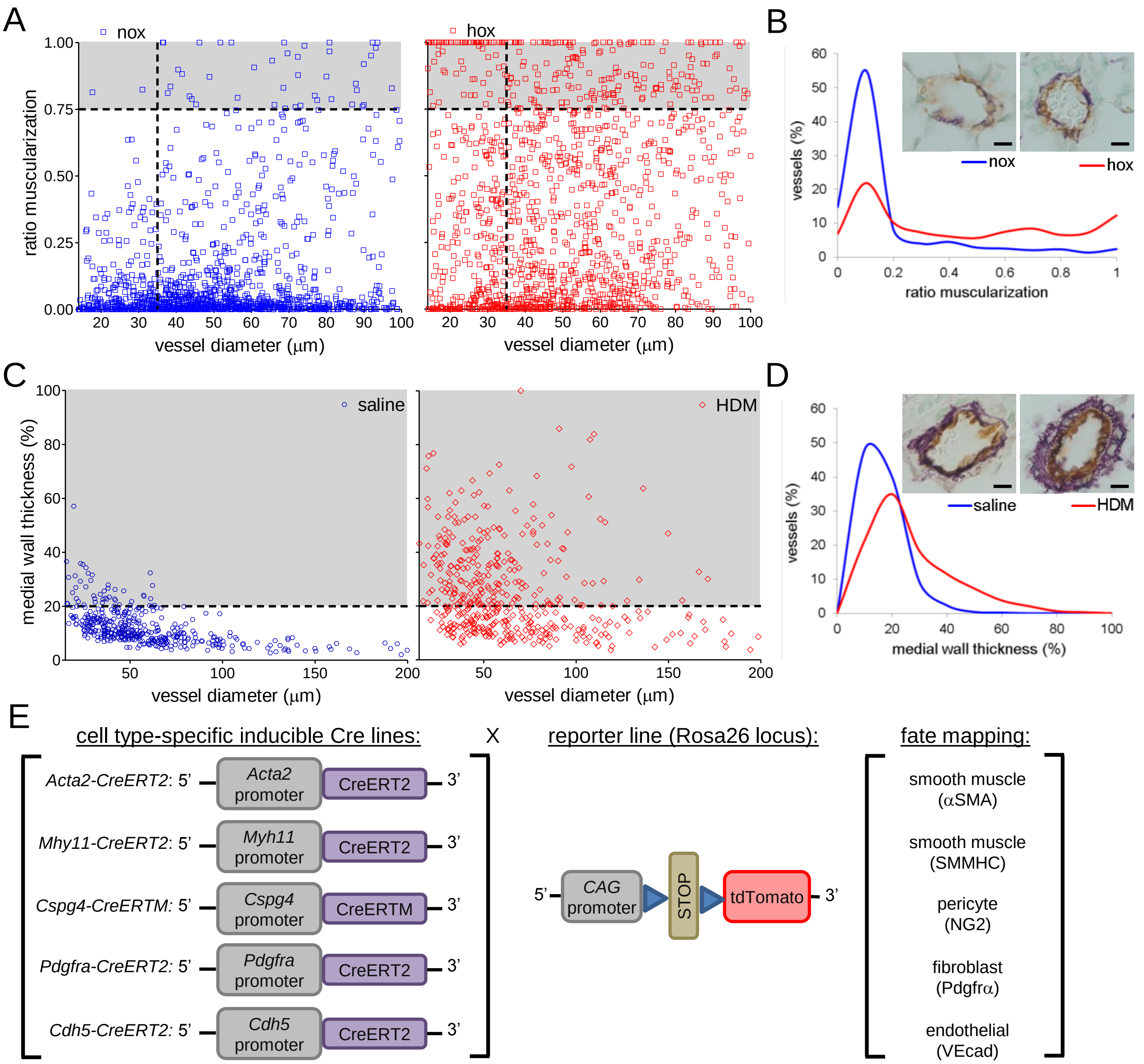

Supplementary Figure S1. Assessment of pulmonary vascular remodeling in mice. (A) Ratio muscularization (αSMA-positive proportion of vessel wall circumference), control (nox), n=1281; chronic hypoxia (hox)-exposed mice, n=1351 vessels. Majority of vessels in controls with diameter <35 µm are not fully muscularized (gray region) . (B) Distribution of vessels according to ratio muscularization. (C) Medial wall thickness of peribronchial/alveolar duct arteries (n=441 control, n=449 HDM-exposed mice). Majority of arteries in controls have wall thickness bellow 20%. (D) Distribution of arteries according to medial wall thickness. Inserts (B;D) show representative double IHC staining of vessels against αSMA (purple) and von Willebrand factor (brown). Scale bar=10 µm. (E) Generation of cell-type specific conditional tdTomato reporter transgenic mice under αSMA (*Acta2-CreERT2*), smooth muscle myosin heavy chain (*Myh11-CreERT2*), neural/glial antigen 2 (*Cspg4-CreERTM*), platelet-derived growth factor receptor alpha (*Pdgfra-CreERT2*), and vascular-endothelial cadherin (*Cdh5-CreERT2*) promoter control.

Supplementary Figure S2

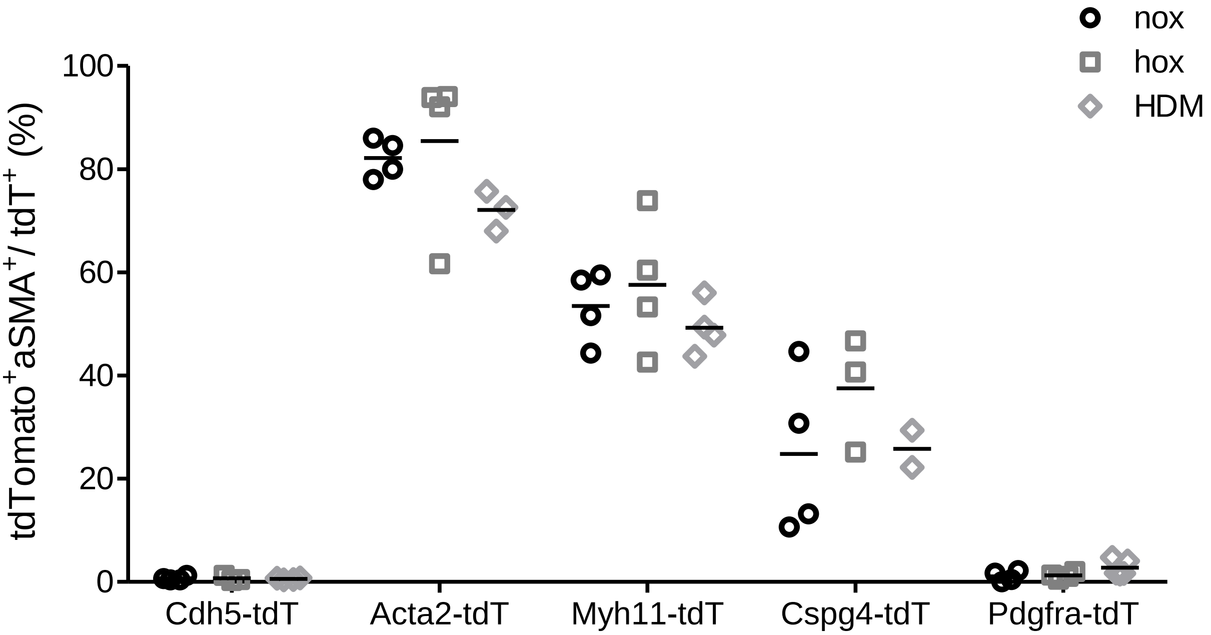

Supplementary Figure S2. Flow cytometric analysis of lineage labeling. Percentage of tdTomato$^+$$\alpha$SMA$^+$ cells within total tdTomato$^+$ cells from lungs of normoxia and chronic hypoxia-exposed mice (n=2-5 mice/group). Each point represents a single animal and with line depicting mean value.

Supplementary Figure S3

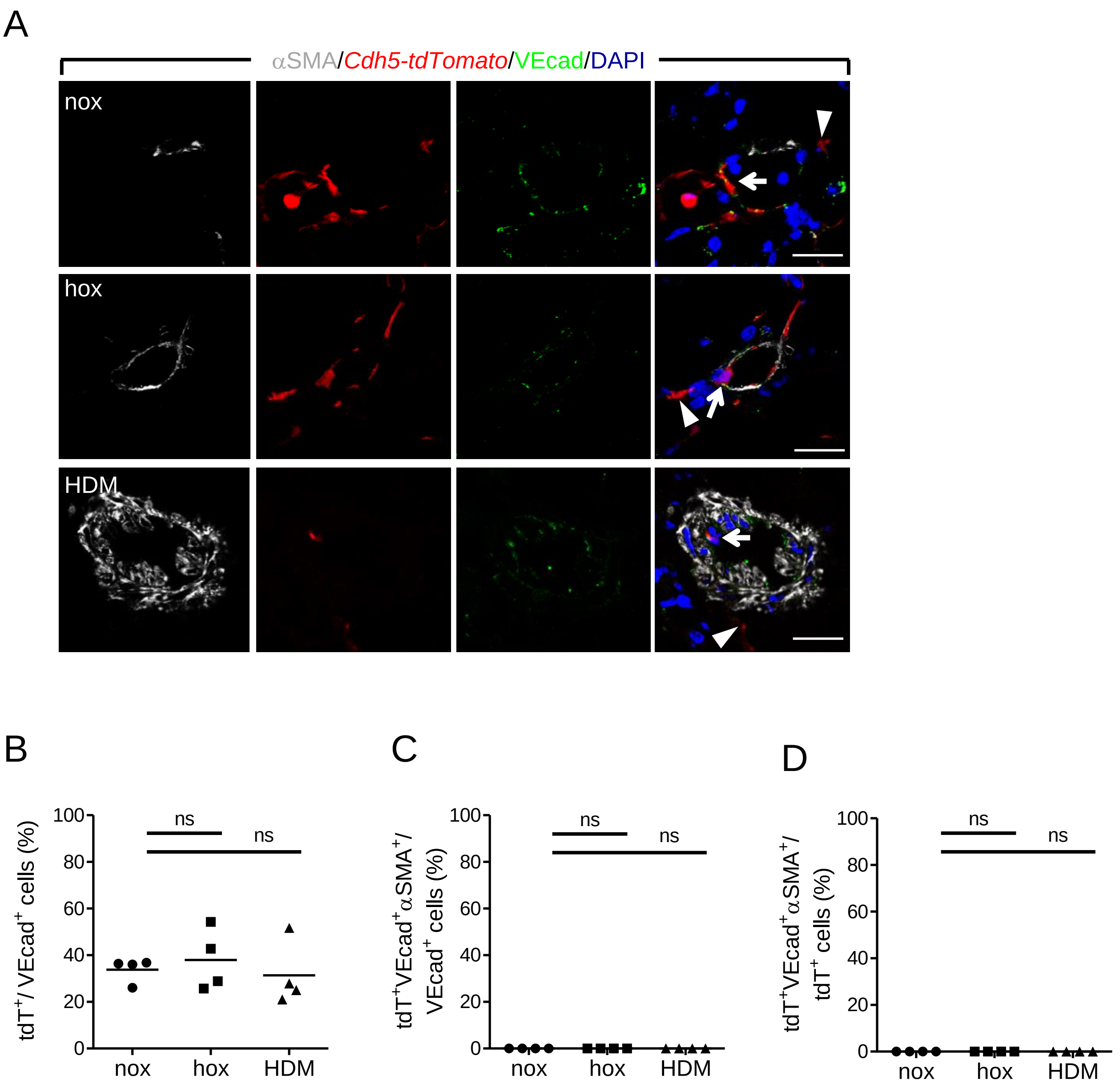

Supplementary Figure S3. Labeling efficiency of Cdh5-tdTomato mouse line. (A) Representative laser scanning confocal micrographs for the assessment of co-localization of VEcad and αSMA-immunostaining with Cdh5-tdTomato. Arrows depict tdTomato-VEcad double positive cells, while tdTomato single positive cells are depicted with arrowheads. White scale bar depicts 20 μm. (B) Quantification of tdTomato labeling efficiency. (C) Percentage of VEcad+ cells co-labeled with tdTomato and αSMA. (D) Percentage of Cdh5-tdTomato+ cells co-labeled with VEcad and αSMA. Each point represents a measurement based on at least 60 VEcad+ cells in a single animal.

Supplementary Figure S4

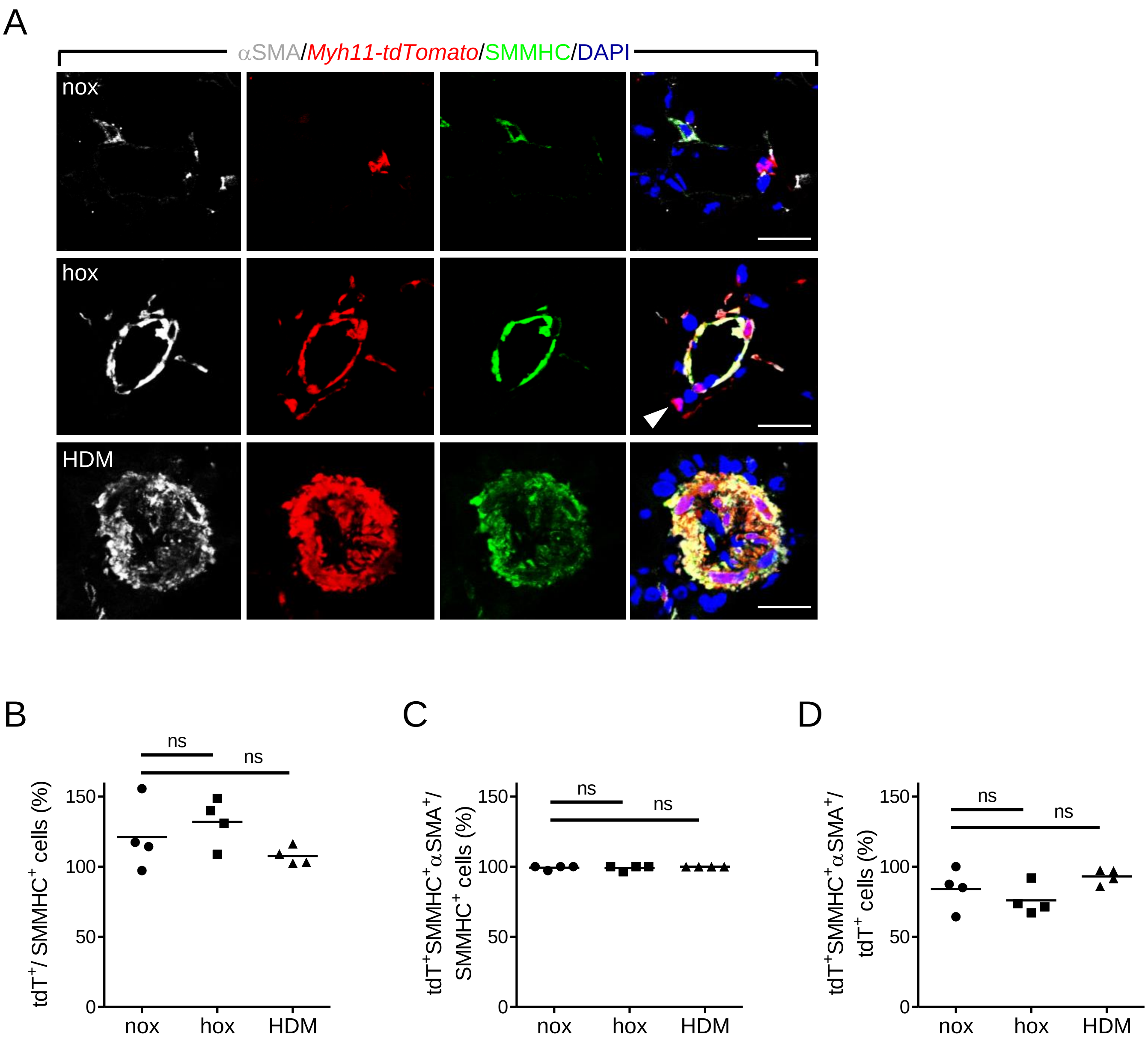

Supplementary Figure S4. Labeling efficiency of Myh11-tdTomato mouse line. (A) Representative laser scanning confocal micrographs for the assessment of co-localization of SMMHC and αSMA-immunostaining with Myh11-tdTomato. Arrows depict tdTomato-SMMHC double positive cells, while tdTomato single positive cells are depicted with arrowheads. White scale bar depicts 20 μm. (B) Quantification of tdTomato labeling efficiency. (C) Percentage of SMMHC+ cells co-labeled with tdTomato and αSMA. (D) Percentage of Myh11-tdTomato+ cells co-labeled with SMMHC and αSMA. Each point represents a measurement based on at least 40 SMMHC+ cells in a single animal.

Supplementary Figure S5

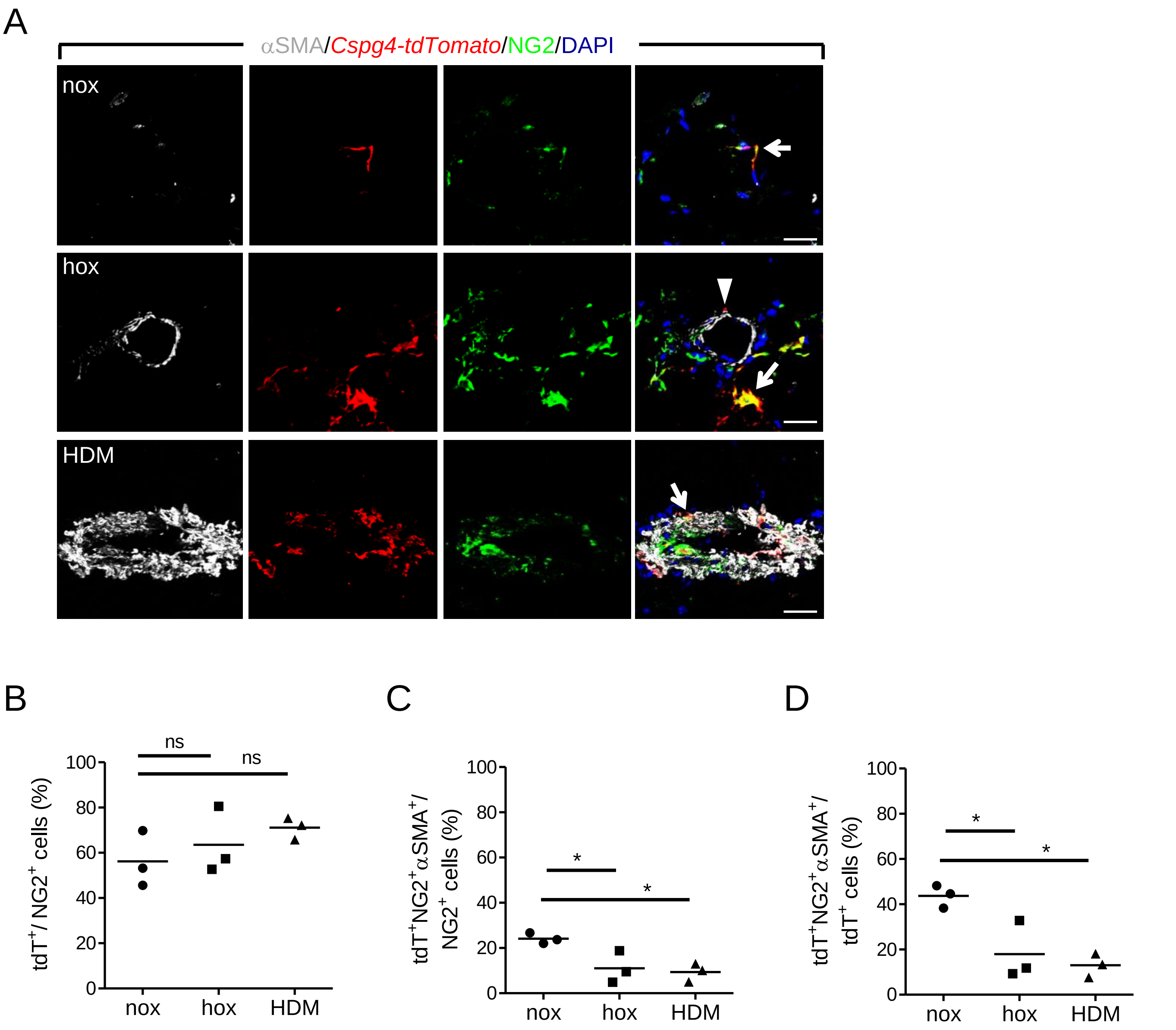

Supplementary Figure S5. Labeling efficiency of Cspg4-tdTomato mouse line. (A) Representative laser scanning confocal micrographs for the assessment of co-localization of NG2 and αSMA-immunostaining with Cspg4-tdTomato. Arrows depict tdTomato-NG2 double positive cells, while tdTomato single positive cells are depicted with arrowheads. White scale bar depicts 20 μm. (B) Quantification of tdTomato labeling efficiency. (C) Percentage of NG2+ cells co-labeled with tdTomato and αSMA. (D) Percentage of Cspg4-tdTomato+ cells co-labeled with NG2 and αSMA. Each point represents a measurement based on at least 110 NG2+ cells in a single animal.

Supplementary Figure S6

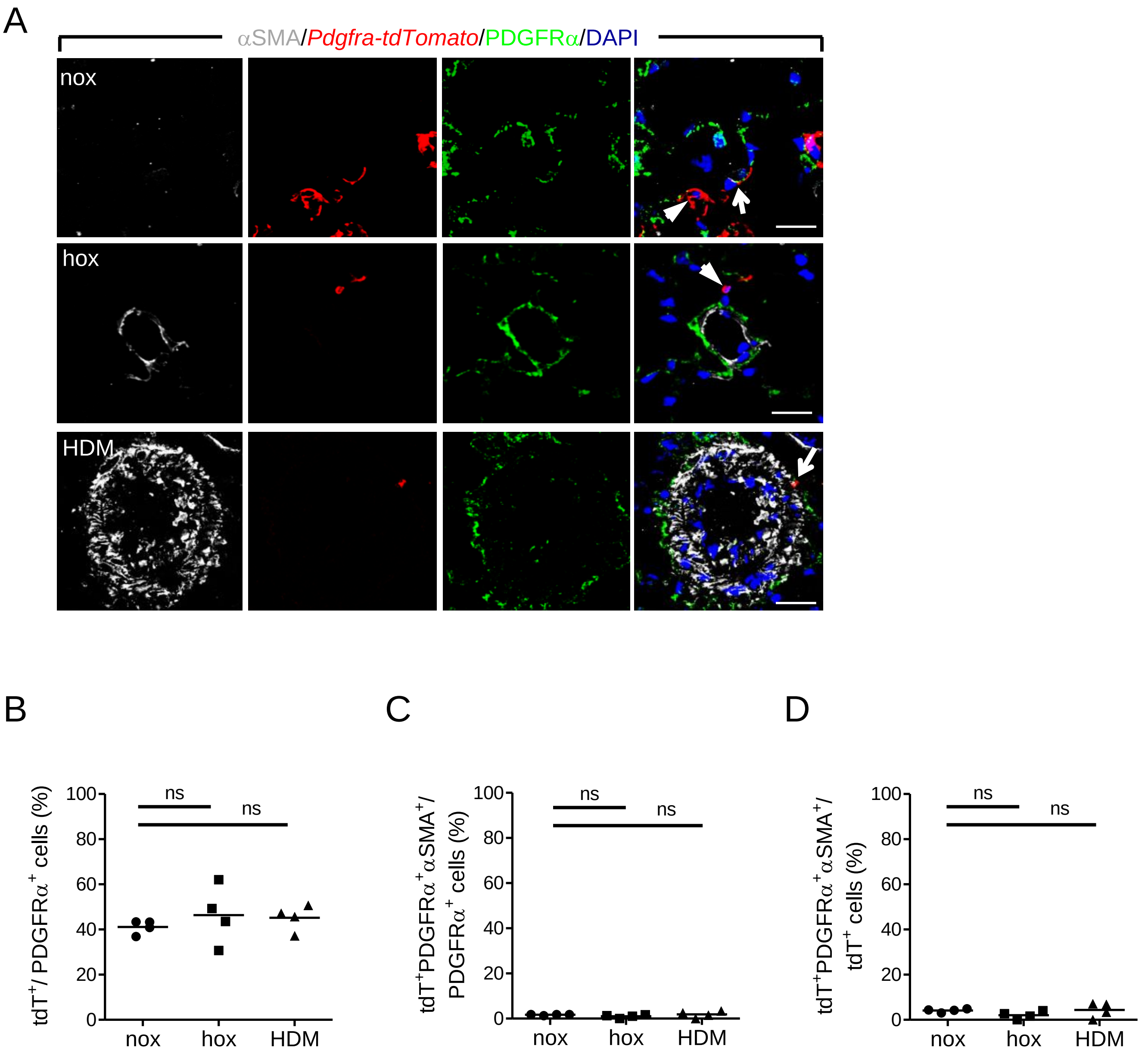

Supplementary Figure S6. Labeling efficiency of Pdgfra-tdTomato mouse line. (A) Representative laser scanning confocal micrographs for the assessment of co-localization of PDGFRαand αSMA-immunostaining with Pdgfra-tdTomato. Arrows depict tdTomato-PDGFRα double positive cells, while tdTomato single positive cells are depicted with arrowheads. White scale bar depicts 20 μm. (B) Quantification of tdTomato labeling efficiency. (C) Percentage of PDGFRα+ cells co-labeled with tdTomato and αSMA. (D) Percentage of Pdgfra-tdTomato+ cells co-labeled with Pdgfrαand αSMA. Each point represents a measurement based on at least 75 Pdgfrα+ cells in a single animal.

Supplementary Figure S7

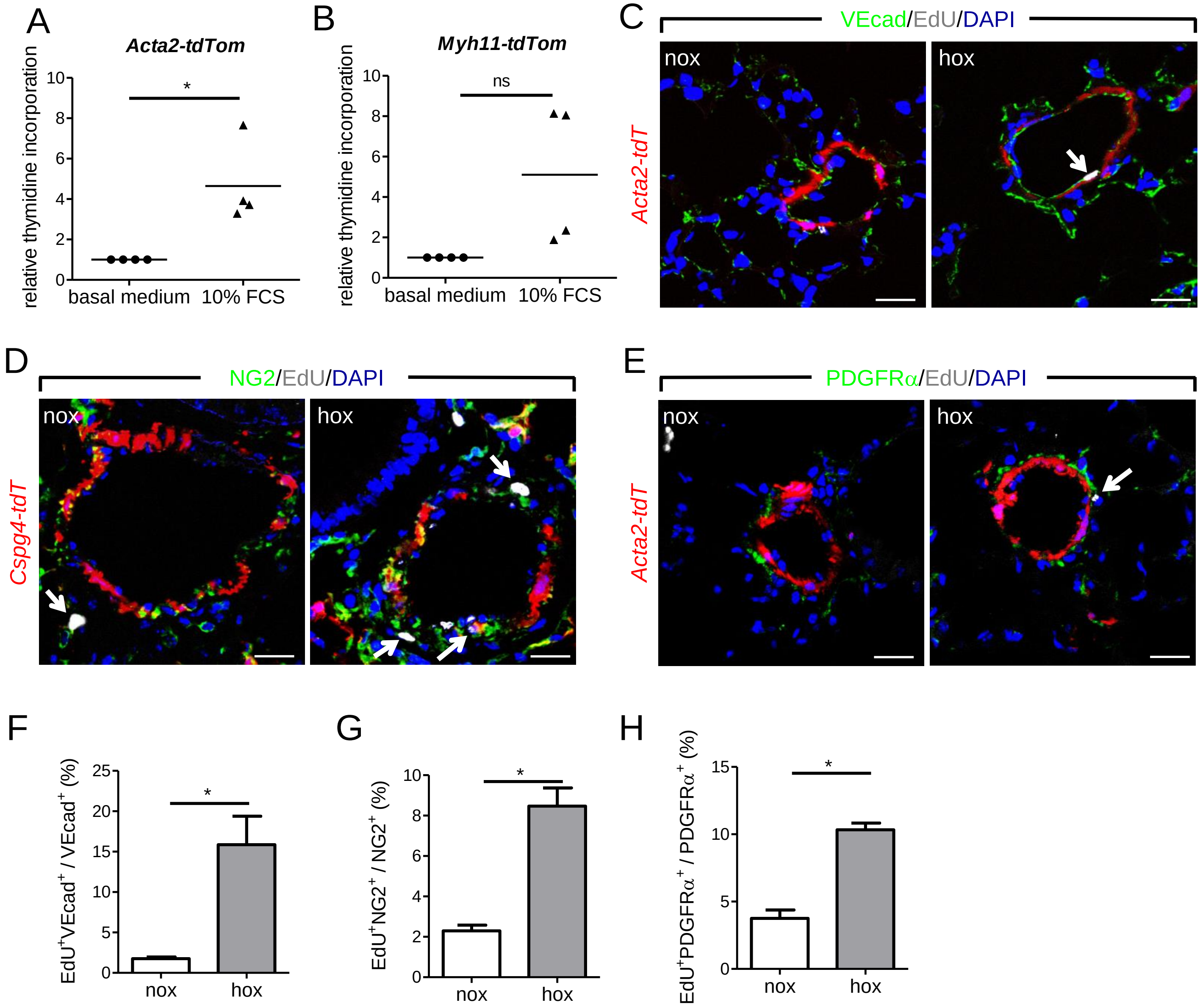

Supplementary Figure S7. Proliferative capacity of (peri)vascular resident cells. (A) Acta2-tdTomato+ or (B) Myh11-tdTomato+ cells were isolated from the main left pulmonary artery tissue pieces (n=4 mice) and their *in vitro* proliferative response to 10% fetal calf serum (FCS) measured using thymidine incorporation assay. (C-E) Representative images showing localization of proliferation label (EdU) in nuclei (white arrows) with VEcad, NG2 and PDGFRα immunostaining in peribronchial arteries from *Acta2-tdTomato* (C,E) or *Cspg4-tdTomato* reporter mice. White scale bar depicts 20 μm. Percentage of VEcad- (F), NG2- (H), and PDGFRα- (H) positive (peri)vascular cells labeled with EdU (n=2-3 mice/group, n=55-135 nuclei/mouse).

Supplementary Figure S8

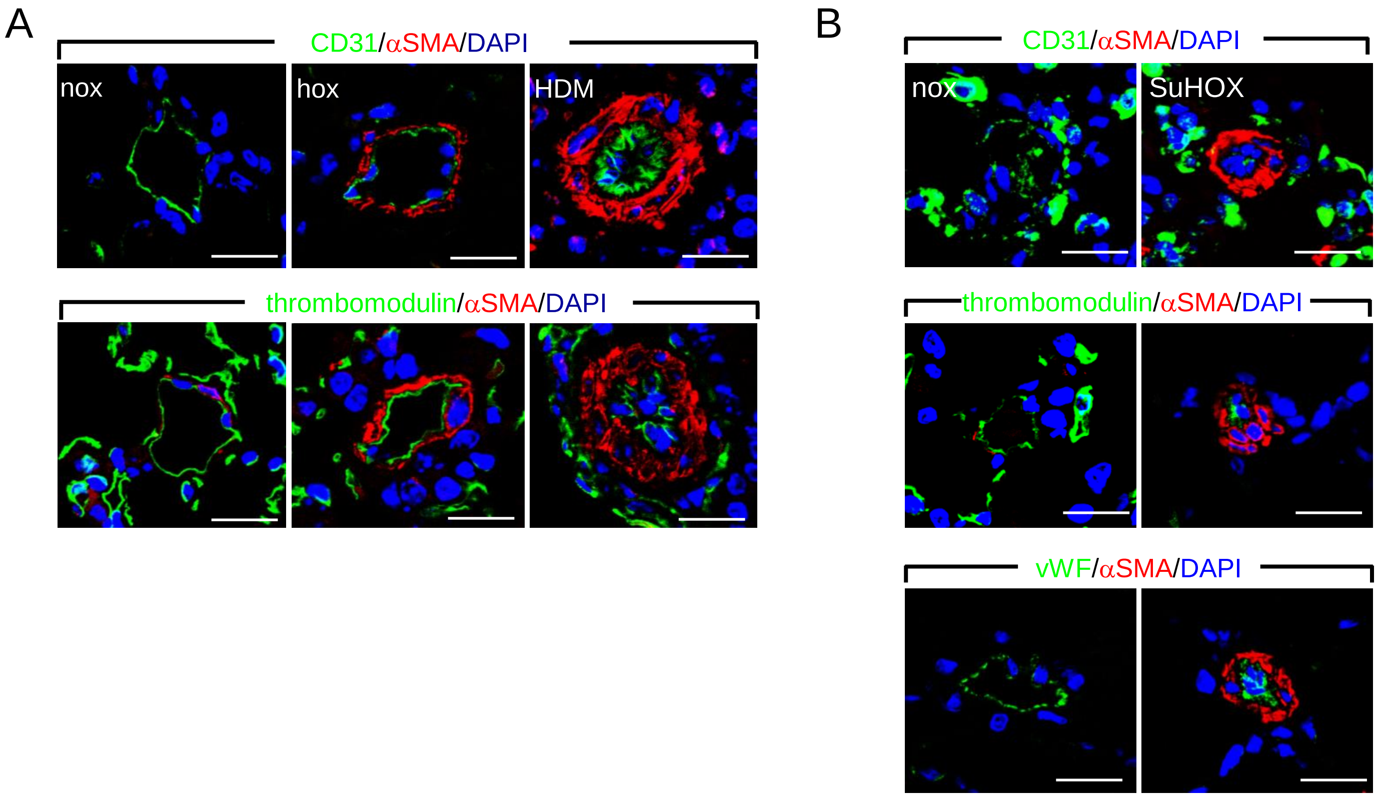

Supplementary Figure S8. Localization of lineage markers in rat pulmonary arteries. Representative immunofluorescent co-staining of alpha smooth muscle actin (αSMA), CD31, thrombomodulin, and von Willebrand factor (vWF) on (A) mouse (normoxia/saline-nox, chronic hypoxia-hox, house dust mite-HDM) and (B) rat (nox, SU5416/hypoxia) lung samples. 4',6-diamidino-2-phenylindole (DAPI) was used as nuclear counterstain. White scale bar depicts 20 µm.

Supplementary Figure S9

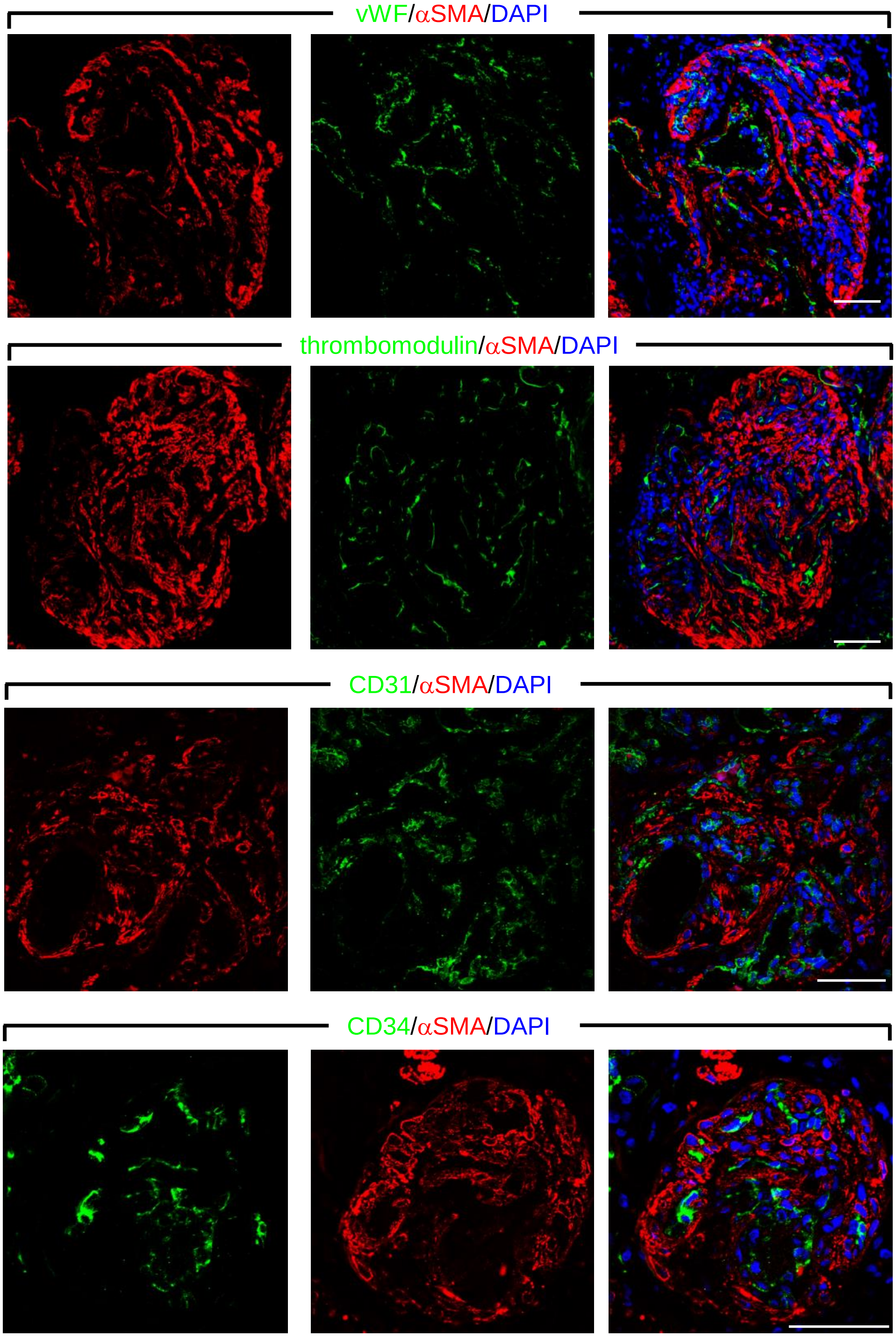

Supplementary Figure S9. Localization of lineage markers in plexiform lesions from IPAH patients. Representative immunofluorescent co-staining of alpha smooth muscle actin (αSMA), von Willebrand factor (vWF), thrombomodulin, CD31, and CD34 on plexiform lesions. 4',6-diamidino-2-phenylindole (DAPI) was used as nuclear counterstain. White scale bar depicts 50 μm.
