# Supplementary material for: Resident cell lineages are preserved in pulmonary vascular remodeling
Source: J Pathol. 2018 Mar 9;244(4):485–98. doi: 10.1002/path.5044 (PMC5903372; doi:10.1002/path.5044)
Supplement: Supplementary file 3 — Table S1. Patient characteristics Table S2. Antibody details Table S3. Primer sequences [file PATH-244-485-s003.docx]

**Table S1.** Patient characteristics

| **Patient** | **Age** | **Sex** | **Mean pulmonary artery pressure (mmHg)** | **Pulmonary vascular resistance (dyn s/cm^5^)** | **Cardiac index (l/min/m^2^)** | **Analysis** |
| --- | --- | --- | --- | --- | --- | --- |
| IPAH 1 | 37 | f | 81 | 2500 | 1.2 | Microscopy |
| IPAH 2 | 25 | f | 87 | NA | 1.9 | Microscopy |
| IPAH 3 | 50 | m | 70 | 720 | 2.1 | Microscopy |
| IPAH 4 | 52 | f | 70 | 1371 | 1.6 | Microscopy |
| IPAH 5 | 38 | f | 102 | 190 | NA | Microscopy |
| IPAH 6 | 27 | f | 70 | NA | 2.5 | Microscopy |
| IPAH 7 | 42 | f | 72 | 1360 | 2.12 | Flow cytometry |
| IPAH 8 | 41 | f | 71 | NA | NA | Flow cytometry |
| IPAH 9 | 38 | f | 54 | NA | NA | Microscopy |
| ILD-PH 1 | 58 | m | 46 | NA | NA | Flow cytometry |
| ILD-PH 2 | 53 | f | 39 | NA | NA | Flow cytometry |
| ILD-PH 3 | 55 | m | 33 | NA | NA | Flow cytometry |
| ILD-PH 4 | 60 | m | 32 | NA | NA | Flow cytometry |
| donor 1 | 47 | m | NA | NA | NA | Microscopy |
| donor 2 | 50 | f | NA | NA | NA | Microscopy |
| donor 3 | 24 | f | NA | NA | NA | Microscopy |
| donor 4 | 42 | f | NA | NA | NA | Microscopy |
| donor 5 | 73 | f | NA | NA | NA | Microscopy |
| donor 6 | 57 | f | NA | NA | NA | Flow cytometry |
| donor 7 | 58 | f | NA | NA | NA | Flow cytometry |
| donor 8 | 58 | f | NA | NA | NA | Flow cytometry |

NA = not available.

**Table S2.** Antibody details

| **Antibody** | **Company** | **Order#** | **Species** | **Dilution** |
| --- | --- | --- | --- | --- |
| SMMHC | Abcam | ab53219 | Rabbit | 1:50 |
| NG2 | Abcam | 129051 | Rabbit | 1:500 |
| VEcad | R&D Systems | AF1002 | Goat | 1:100 |
|  | Santa Cruz | SC-9989 | Mouse | 1:100 |
| PDGFRα | R&D Systems | AF1062 | Goat | 1:500 |
|  | Cell Signaling | 3174S | Rabbit | 1:1000 |
| αSMA | Everest | EB06450 | Goat | 1:200 |
|  | Sigma | F3777/ C6198 | Mouse | 1:200 |
| vWF | Dako | A0082 | Rabbit | 1:500 |
| CD31 | Cell Signaling | 77699S | Rabbit | 1:100 |
|  | Abcam | Ab32457 | Rabbit | 1:100 |
| Thrombomodulin | R&D Systems | AF3894 | Goat | 1:100 |
| CD34 | Biorbyt | orb27549 | Rabbit | 1:100 |

**Table S3.** Primer sequences

| **Primer name** | **Accession number** | **Forward sequence** | **Reverse sequence** |
| --- | --- | --- | --- |
| VWF | NM_000552.4 | GGAGCATGTACAGCTTTGCG | CCCAAGATACACGGAGAGGC |
| VEcad | NM_001795.4 | GCCCATGAAGCCTCTGGATTA | GTATCGGAGGTCGATGGTGG |
| CD31 | NM_000442.4 | TCCGTTGCGAATCGATCAGT | ACGTCTTCAGTGGGGTTGTC |
| FVIII | NM_000132.3 | TTTGCCACCTGGTCTCCTTC | TGGATTATTCACCTGAGGTCTCC |
| αSMA | NM_001141945.2 | CTTTCAGCTTCCCTGAACACC | TTACAGAGCCCAGAGCCATT |
| SMMHC | NM_001040113.1 | AAGCAGCTTCTACAAGCAAAC | TTGGCTCCCACGATGTAACC |
| NG2 | NM_001897.4 | CACAGAGGAACCCTGGATGG | TTCAGCGAGAGGAGCACTTG |
| Pdgfra | NM_006206.5 | TAGTGCTTGGTCGGGTCTTG | TTCATGACAGGTTGGGACCG |
| Pdgfrb | NM_002609.3 | GTTCAAAGACAACCGCACCC | CAGCTCTGACACATACCGGG |
| Fibronectin | NM_212482.2 | CTGCAGGTCCAGATCAAACAG | CCAGTCCTTTAGGGCGATCAA |
| S100A4 | NM_019554.2 | CTAAAGGAGCTGCTGACCCG | TCCCTGTTGCTGTCCAAGTTG |
| Cyclin D1 | NM_053056.2 | AGTGGAAACCATCCGCCG | TCTGTTCCTCGCAGACCTCCA |
| b2m | NM_004048.2 | CCTGGAGGCTATCCAGCGTACTCC | TGTCGGATGGATGAAACCCAGACA |
| pbdg | NM_000190.3 | TCGGAGCCATCTGCAAGCGG | GCCGGGTGTTGAGGTTTCCCC |
